# Structure Determination of Tegoprazan((*S*)-4-((5,7-difluorochroman-4-yl)oxy)-*N*,*N*,2-trimethyl-1*H*-benzo[*d*]imidazole-6-formamide) Polymorphs A and B by Laboratory X-Ray Powder Diffraction

**DOI:** 10.3390/molecules30071538

**Published:** 2025-03-30

**Authors:** Seah Ryu, JooHo Lee, Jason Kim, Tokutaro Yamaguchi

**Affiliations:** J2Hbiotech Inc. Corp, #210, B dong, 142-10, Saneop-ro 156beon-gil, Gwonseon-gu, Suwon-si 16648, Gyeonggi-do, Republic of Korea; fbtpdk0917@gmail.com (S.R.); shadow@j2hbio.com (J.L.); jsbach@j2hbio.com (J.K.)

**Keywords:** tautomerism, desmotropism, powder X-ray diffraction, SDPD, Tegoprazan, DFT-D

## Abstract

Tegoprazan is a potassium ion-competitive acid blocker (P-CAB) and a novel inhibitor of gastric acid secretion. The compound exists in two crystalline polymorphs, A and B, whose structures had not previously been reported. In this study, both polymorphs were analyzed by liquid- and solid-state NMR, revealing identical tautomeric states. Using this information, the crystal structures were determined from laboratory powder X-ray diffraction data by simulated annealing and Rietveld refinement. Both forms were found to crystallize in the monoclinic space group P2_1_, with Z = 4 and two independent molecules in the asymmetric unit (Z′ = 2). To assess the stability and reliability of the refined structures, we attempted geometry optimization and vibrational analysis using DFT-D methods. However, due to the high conformational complexity of Z′ = 2 systems, these calculations failed to converge or produced imaginary frequencies. Instead, single-point energy calculations were performed on the refined models. The resulting relative energy differences, together with solubility data, van’t Hoff enthalpies, and DSC profiles, consistently indicated that Polymorph A is more stable than Polymorph B. These results highlight the challenges of structure validation via DFT-D for complex molecular crystals and demonstrate the value of integrating experimental and computational approaches for polymorph characterization.

## 1. Introduction

Tegoprazan is a potassium-competitive acid blocker (P-CAB) developed for treating acid-related gastrointestinal disorders [1,2]. While proton pump inhibitors (PPIs) have been widely used for gastroesophageal reflux disease (GERD), P-CABs represent a newer class of powerful and selective inhibitors of gastric acid secretion. P-CABs work more quickly than PPIs and can maintain gastric acid pH control for longer periods. The effectiveness of Tegoprazan stems from its slow release from gastric glands, making it effective regardless of gastric acid levels or food intake. Excessive gastric acid secretion is linked to several diseases, including GERD, also known as reflux esophagitis [3,4], which has led to a growing demand for these agents and extensive research into their use. It has been found to be useful as a treatment for acid-related gastrointestinal disorders; also, two polymorphs of Tegoprazan, A and B, have been reported [5,6].

In pharmaceutical product development and maintenance, unexpected demands for quality improvements often arise, requiring rapid, yet effective, responses. This includes investigating previously unnecessary aspects of even older products, driven by new discoveries of phenomena or therapeutic effects. In such cases, the development of cost-effective, straightforward, and rapid methodologies becomes essential.

As a case in point, the stability of the two Tegoprazan polymorphs was evaluated under accelerated conditions (temperature: 40 ± 2 °C, relative humidity: 75 ± 5 HR%) over an 8-week period, with observations for any significant changes conducted using DSC (Differential Scanning Calorimetry) and XRD (X-ray diffraction). Significant changes were observed in the eighth week, with Polymorph B appearing to undergo a phase transition to Polymorph A (Figure S34). No changes were observed in Polymorph A. This phase transition phenomenon is expected to have significant implications in pharmacology, as well as in quality control, formulation manufacturing, and other areas. Therefore, it is essential to elucidate the details of this phenomenon. For quantitative phase analysis and similar studies, knowledge of the crystal structure is crucial. However, the crystal structures of Polymorphs A and B remain unresolved. In response to this need, we explored a practical approach to crystal structure analysis by utilizing readily available powder samples, laboratory-scale XRD instruments, and cost-efficient Rietveld refinement software specifically EXPO2014 (expo version 1.22.11). The known method of SDPD (Structure Determination from Powder Diffraction) was considered as one potential solution to address these challenges. While X-ray diffraction often utilizes synchrotron radiation or other advanced accelerator-based facilities, the crystal structures of Tegoprazan Polymorphs A and B were analyzed using laboratory-based X-ray diffraction (XRD) equipment and powdered samples. This approach was chosen for the following reasons:

Single crystals are commonly used in crystallographic analysis to determine crystal structures, with X-ray diffraction and neutron diffraction being the most widely employed methods. However, they are rarely directly used in pharmaceutical manufacturing. Instead, powders are commonly used as raw materials in pharmaceutical formulations. Additionally, physicochemical studies of crystal polymorphs, such as screening and solubility measurements, often focus on specific crystalline powders. Since crystal transitions frequently occur as solvent-mediated transformations from less stable to more stable forms, in situ X-ray diffraction measurements of phase transitions are desirable for studying crystallographic stability to facilitate rapid crystallographic analysis. Consequently, the aim was to obtain crystallographic data relevant to pharmaceutical formulations not from single crystals but from the powder form of the production material, emphasizing equivalency. Measurements were conducted using a laboratory powder X-ray diffractometer, which is more accessible compared to synchrotron radiation or similar advanced facilities.

In the course of crystal structure analysis, the tautomeric state of Tegoprazan, specifically the position of the NH group within the benzimidazole skeleton, is a critical factor influencing the accuracy of the analysis. Tautomerism is a well-documented phenomenon in imidazole structures, where the asymmetry of the benzimidazole moiety can result in the formation of structural isomers depending on the position of the NH group [7,8,9,10,11,12]. Therefore, it is imperative to consider the unique structural characteristics of Tegoprazan, particularly its tautomerism, to ensure precise and reliable crystallographic determination.

Liquid-state NMR studies have shown that two tautomers exist when an asymmetric benzimidazole moiety is present. These tautomers are indistinguishable by ^1^H and ^13^C-NMR at room temperature because tautomerization occurs rapidly in nonpolar solvents such as CDCl_3_. In contrast, in polar solvents such as DMSO-*d6*, the tautomerization rate is slower, allowing the chemical shifts of the two tautomers to become observable due to changes in the position of the NH group in the imidazole ring [13,14,15]. When the benzimidazole moiety is asymmetric and bonds to an inducible element, such as oxygen or halogen, ^13^C-NMR often shows significant differences in the chemical shifts between tautomers. Tautomerism in the solid state is extremely rare, and it is generally assumed that the position of the NH group remains fixed during crystallization from the solution phase. Therefore, it is crucial to determine whether the crystallized form consists of one or both tautomers.

**Figure 1:**
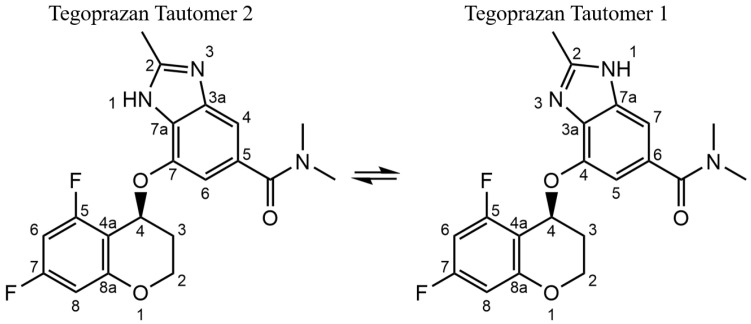
Difference in the position of the NH group of Tegoprazan due to tautomerization indicates the presence of two tautomeric isomers, and this structural difference can be confirmed. It is important to note that the atom labels for Tegoprazan follow IUPAC nomenclature, which differs from the labels used in NMR analysis and crystallographic analysis. The distinction in the NMR analysis is made to avoid confusion as the same numbering is used for both the benzimidazole and chromane moieties.

The differences in the chemical shifts of each tautomer in non-protonic solvents were utilized to investigate which tautomer corresponds to the crystallized form of Tegoprazan. To achieve this, solid-state ^13^C-NMR chemical shifts were compared to the liquid-state ^13^C-NMR chemical shifts at room temperature and in DMSO-*d6*. For this comparison, structural analysis of each tautomer via 1D (^1^H and ^13^C) and 2D (COSY (Correlation Spectroscopy), ROESY (Rotating Frame Overhauser Enhancement Spectroscopy), HSQC (Heteronuclear Single Quantum Coherence), and HMBC (Heteronuclear Multiple Bond Coherence)) NMR measurements were required, including the identification of each element (^1^H, ^13^C, and ^19^F) of the tautomers, as shown in Figure 1. The identification was carried out by measuring a mixture of each tautomer in DMSO-*d6* solution at room temperature. (This mixture can be prepared simply by dissolving Polymorph A in DMSO-*d6*. If this approach is unsuccessful, the mixture can be confirmed by adding a trace amount of trifluoroacetic acid, commonly used as a standard reagent for ^19^F NMR).

The analyses revealed that the position of the hydrogen in the NH group, which could not be accurately determined by X-ray diffraction, is identical in both Polymorphs A and B of Tegoprazan, corresponding to Tautomer 1, as shown in Figure 1. These results show that the crystal structures of Polymorphs A and B are not desmotropic [16,17].

Building on this, the structure of Tautomer 1 was utilized as the basic structure for Polymorphs A and B. Their crystal structures were determined using X-ray diffraction data collected with a laboratory powder X-ray diffractometer, employing simulated annealing followed by Rietveld refinement. Both polymorphs were found to crystallize in the monoclinic space group P2_1_ with Z = 4, where the asymmetric unit contains two symmetrically independent molecules.

In this study, we attempted to validate the experimentally refined crystal structures of two Tegoprazan polymorphs using dispersion-corrected density functional theory (DFT-D) by comparing optimized and experimental geometries via RMSCD calculations. However, due to severe computational limitations associated with Z′ > 1 systems—including convergence failures and imaginary frequencies—we adopted a single-point energy approach based on refined Rietveld models. The resulting energy differences, in conjunction with solubility and DSC data, consistently identified Polymorph A as the more stable form. These findings demonstrate both the practical limitations of DFT-D optimization for complex crystal systems and the complementary role of experimental and computational thermodynamic analyses.

## 2. Results and Discussion

### 2.1. Structural Analysis of Tegoprazan Using NMR Spectroscopy

#### 2.1.1. Analysis of Tautomerization in Tegoprazan

Tegoprazan contains a benzimidazole moiety, and its tautomerization exchange rate in DMSO-*d6* is relatively slow, likely due to steric and electronic factors inherent to its molecular structure. This slow exchange rate impacts NMR analysis by allowing distinct peaks for Tautomer 1 and Tautomer 2 to appear in the spectrum, making the precise determination of coupling constants challenging as overlapping signals and broadened peaks complicate spectral interpretation. This allows both Tautomer 1 and Tautomer 2 to be observed simultaneously in the same NMR spectrum. Notably, the two tautomers exhibit distinct chemical shifts for corresponding carbon and hydrogen atoms due to variations in their electronic environments and bonding arrangements, enabling clear differentiation and detailed structural analysis of Tegoprazan in DMSO-*d6*. In this study, each tautomer present in DMSO-*d6* was analyzed simultaneously in a mixed state, and each peak was assigned to the corresponding tautomer. NMR measurements were performed at a temperature of 23 ± 3 °C.

The differences between the Tegoprazan Tautomers 1 and 2 were confirmed by ^1^H-NMR and ^13^C-NMR in DMSO-*d6*, as shown in Tables S1 and S2. These chemical shifts and ^1^H-^1^H spin-spin coupling constants were validated using 1D (^1^H and ^13^C) NMR, as well as 2D techniques, including COSY, ROESY, HSQC, and HMBC. In an aliphatic six-membered ring, the coupling constant of hydrogens bonded to adjacent carbons differs significantly depending on their spatial orientation. When the hydrogens are in an axial–axial or geminal relationship, the coupling constant ranges from 7 to 15 Hz, which is larger than the 1.5–5 Hz range observed for equatorial–equatorial or axial–equatorial hydrogens. This difference results in distinctive splitting patterns in the ^1^H-NMR spectrum. In the case of Tegoprazan, the aliphatic carbons 2 and 3 in the chromane moiety exhibit hydrogens bonded to adjacent carbons that present triplet-like splitting patterns when in an axial orientation. Due to tautomerism, with Tautomer 1 and Tautomer 2 coexisting, slight differences in chemical shifts were observed. The differences in value were similar to those of axial–axial or geminal coupling constants. This overlap of peaks results in a quartet-like pattern. In the chromane moiety, such peaks were indeed identified and attributed to axial hydrogens. In contrast, equatorial hydrogens, with their smaller coupling constants, exhibit doublet-like splitting patterns. The hydrogens bonded to carbons 2 and 3 in the chromane moiety are easily distinguishable based on their chemical shifts, which vary significantly depending on whether the carbon is also bonded to oxygen. This difference further facilitates assignment (see Supplementary Materials, Figures S1–S27). C2 of the chromane moiety adjacent to oxygen was downfield from C3, appearing at about 62 ppm, and hydrogen bonded to C2 was also downfield, appearing at about 4.3 ppm. The hydrogens of the aliphatic carbons in the chromane moiety of Tegoprazan in DMSO-*d6* were labeled as multiplets (Tables S1 and S2) due to the complications introduced by slow tautomerism, which causes overlapping peaks and hinders the precise determination of coupling constants.

The most easily identifiable carbons and hydrogens in Tegoprazan are the chiral carbon (C4) and hydrogen (H4) of the chromane moiety. This chiral center is a methine group, consisting of a single carbon atom bonded to a hydrogen atom and an oxygen atom. The chemical shifts of these nuclei were confirmed using HSQC spectra of the positive peaks, whose integrals correspond to one proton, showing carbon shifts at 63.05 ppm and 62.59 ppm and hydrogen shifts at 6.18 ppm and 5.88 ppm, respectively. These two pairs of signals, unique within the structure, served as reference points for assigning the chemical shifts of other carbons and hydrogens in each tautomer. Cross-peaks from COSY, ROESY, HSQC, and HMBC spectra were utilized to identify and assign the chemical shifts, starting from these pairs associated with the chiral center. Fluorine assignments were further clarified using ^13^C-^19^F HSQC and ^13^C-^19^F HMBC experiments. It must be clarified whether the carbon at the bonding site via oxygen between the chromane and benzimidazole moieties corresponds to the C4 of Tautomer 1 of benzimidazole or the C7 of Tautomer 2.

**Figure 2:**
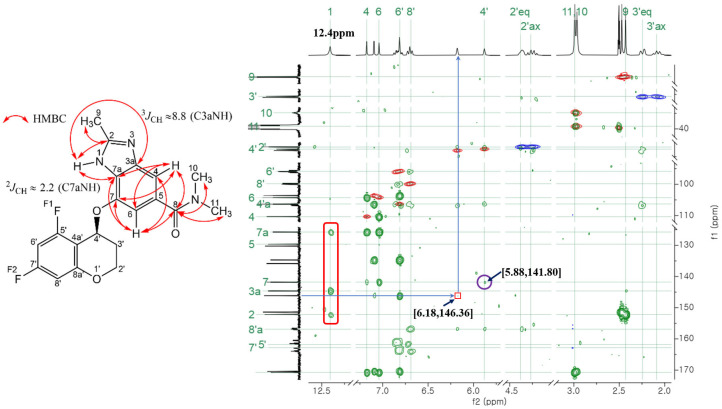
Chart overlaying ^1^H-^13^C HSQC and HMBC spectra of two tautomer mixtures of Tegoprazan measured in DMSO-*d6* solution on a 400 MHz NMR instrument. Starting with 5.88 ppm of hydrogen and 141.80 ppm of carbon, the associated carbon and hydrogen were assigned. In the structure of Tegoprazan shown on the left, the curved arrows indicate the positions of carbons and hydrogens that exhibit long-range coupling, as observed through HMBC cross-peaks. Within the benzimidazole moiety, some hydrogens and carbons were identified based on their correlations, enabling partial assignment of the carbon signals. The cross-peaks enclosed in the red rounded rectangle correspond to the interactions between the hydrogen of the NH group and its associated carbons.

Initially, the hydrogen at 5.88 ppm was used as a reference for assignment as its correlation peak in the HMBC spectrum was relatively more distinct compared to the hydrogen at 6.18 ppm. In the process of attributing the chromane moiety, the hydrogen at 5.88 ppm was found to cross-peak with four carbons in HMBC, three of which were carbons of the chromane moiety. The remaining carbon at 141.80 ppm that could not be attributed to the chromane moiety is the carbon of the benzimidazole moiety, which is bonded to chromane via oxygen. Subsequently, using the carbon at 141.80 ppm as a reference, the majority of the hydrogen and carbon atoms were assigned to the benzimidazole moiety based on the correlation peaks observed in the 2D HMBC and HSQC spectra, as shown in Figure 2. The ^1^H-NMR analysis reveals distinct splitting patterns for the aromatic hydrogens in the benzimidazole and chromane moieties of Tegoprazan. In the benzimidazole moiety, hydrogens on the benzene ring are located at meta positions relative to each other, resulting in long-range coupling constants of 1–3 Hz. Consequently, these hydrogens appear as singlet-like peaks in the spectrum. In contrast, the aromatic hydrogens on the chromane moiety’s benzene ring, despite also being in meta positions, do not exhibit singlet-like peaks due to the influence of fluorine substitution. Fluorine, attached to a carbon adjacent to the hydrogen-bonded carbon, interacts with hydrogens via spin-spin coupling akin to proton–proton interactions. The coupling constants for ortho position (^3^*J*_HF_) interactions are approximately 6–10 Hz, while those for meta position (^4^*J*_HF_) interactions range from 5 to 6 Hz. Thus, the peak shapes for the aromatic hydrogens in the benzimidazole and chromane moieties are readily distinguishable. In the HMBC spectrum, the carbon at 141.80 ppm exhibits two cross-peaks with singlet-like hydrogens, confirming their assignment to the aromatic hydrogens of the benzimidazole moiety. Among these cross-peaks, the stronger one corresponds to hydrogen 6 closer to the carbon at 141.80 ppm. Another one was identified as hydrogen 4. Additionally, these aromatic hydrogens show cross-peaks with the carbonyl carbon of the amide group in the HMBC spectrum. The carbonyl carbon further exhibits cross-peaks with the hydrogens of the methyl group attached to the amide nitrogen, solidifying the structural assignments.

C3a and C7a of the imidazole moiety were identified by correlation with a broad singlet-like hydrogen peak around 12.4 ppm with carbon that cross-peaks with hydrogen 4 and hydrogen 6 of the benzimidazole moiety identified above in the HMBC spectrum. One such cross-peak corresponds to a hydrogen peak at 12.4 ppm, which also showed a stronger correlation with the carbon at 144.62 ppm. The other two cross-peaks were weaker and of similar intensity, suggesting that these are cross-peaks with carbons C2, C3a, and C7a. C2 exhibited a strong cross-peak with the hydrogen peak of the methyl group at 2.43 ppm in the HMBC spectrum. The carbon of this methyl group was identified as C9, the methyl group attached to imidazole.

Here, the studies by Pinto et al. [18] and Nieto et al. [15] were utilized to precisely identify the position of the NH group in the imidazole ring. Pinto et al. reported that the long-range coupling constants between the NH proton and carbon in imidazole are ^2^*J*_CH_ = 2.2 Hz and ^3^*J*_CH_ = 8.8 Hz. Here, the carbon of the benzimidazole moiety attached via oxygen to the chiral central carbon of the chroman moiety was assumed to be C7. In other words, it was assumed to have the structure of Tautomer 2. In Tautomer 2, the carbon C7a would appear upfield compared to C3a, which is not adjacent to a carbon bonded to oxygen. In the HMBC measurement, with the long-range coupling set to 8 Hz, the carbon showing a relatively strong correlation peak corresponding to a three-bond coupling (^3^*J*_CH_ = 8) with the NH proton was identified as carbon 3a, with a chemical shift of 144.62 ppm. The cross-peak between the carbon peak at 125.74 ppm and the NH-group hydrogen in the HMBC spectrum indicates a two-bond coupling (^2^*J*_CH_) that is weaker in intensity than the three-bond coupling at 144.68 ppm (^3^*J*_CH_ = 8.8 Hz). If the number of each atom in Tautomer 2 is fixed and the NH-group hydrogen is bonded to N3 (thus, when the position of the hydrogen in the NH group changes, the numbering changes according to the IUPAC nomenclature), the cross-peak between the upfield carbon (C7a) and the NH-group hydrogen would be the strongest.

On the other hand, the position of the NH group was also determined to be identical using the method proposed by Roslund et al., which distinguishes between ^2^*J*_H,C_ and ^3^*J*_H,C_ by utilizing the sign of the coupling constants (the cross-peak enclosed in the red rounded rectangle in Figure 2; also, see Figure S25) [19,20].

Hence, the hydrogen, carbon, and fluorine identified from the chiral central hydrogen at 5.88 ppm were found to be reasonable identifications when the structure was set to Tautomer 2. Additionally, the NH proton peak at 12.4 ppm was identified as corresponding to the hydrogen of Tautomer 2, and this was consistently observed in both Polymorph A and Polymorph B.

In benzimidazole, when carbon 4 is assigned to the *trans* position and carbon 7 is assigned to the *cis* position relative to the NH group, the chemical shift difference of 2-methylbenzimidazole in DMSO-*d6* is approximately 7 ppm in ^13^C-NMR, with the *trans*-positioned carbon appearing downfield [15]. Similarly, when substituents are bonded to carbons 4 and 7 of the benzimidazole moiety, some differences are observed in the DMSO-*d6* solution. Therefore, the carbon of the benzimidazole moiety, which is bound via oxygen to the chromane moiety in Tautomer 1, is expected to appear downfield from 141.80 ppm, as explained above, since it is in the trans position relative to the NH group. In fact, the related cross-peak between the other chiral central hydrogen peak at 6.18 ppm and the carbon of the benzimidazole moiety bonded via oxygen was verified in the HMBC spectrum to be a very weak cross-peak with a carbon peak at 146.16 ppm. In the case of Tegoprazan, the bonded carbons from the chromane moiety of Tautomer 1 and Tautomer 2 are *trans* and *cis* for the NH group, respectively. The difference in chemical shift was as small as 4.36 ppm compared to 7 ppm for 2-benzimidazole. Subsequently, the remaining unassigned hydrogen, carbon, and fluorine were identified using the same method as described above, with respect to the hydrogen at 6.18 ppm, leading to the elucidation of an additional chromane moiety and benzimidazole moiety. Furthermore, the previously unassigned carbons, such as those at positions 5 or 6, where the amide group is attached, were similarly identified using the relative chemical shift differences based on the differences observed for 2-methylbenzimidazole in DMSO-*d6*, even in the absence of observable correlation peaks.

#### 2.1.2. Identification of the Tautomer for Tegoprazan Polymorphs A and B

To analyze the crystal structure using powder XRD through simulated annealing and Rietveld refinement, it is essential to establish a fundamental structural model. Consequently, a search for the ground-state structure and iterative trial-and-error processes must be conducted. To minimize the number of iterations, it is preferable to exclude structures with low probabilities of existence. For this purpose, it is necessary to investigate the potential of two tautomers that could result in the two polymorphs of Tegoprazan. Solid-state ^13^C-NMR was employed for this verification. The two tautomers differ in the position of the NH group, and their presence in the crystal affects the observed spectral features as follows:

1. If both tautomers coexist within a single crystal, multiple peaks will be observed for each carbon.
2. If only one tautomer exists and the asymmetric unit contains a single molecule, a single peak will appear for each carbon.
3. If two distinct molecules are present, pairs of peaks in close proximity will be observed, resulting in a simpler spectrum compared to that of coexisting tautomers.

This approach allows the assessment of tautomer distribution and contributes to determining the structural model [21,22,23,24,25].

**Figure 3:**
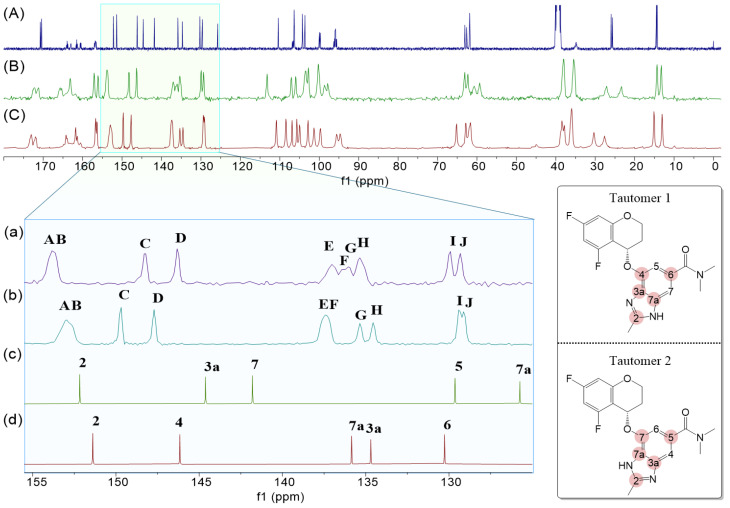
A comparison of the assigned ^13^C-NMR spectra of each tautomer to the solid-state ^13^C-NMR spectra of Tegoprazan Polymorphs A and B is presented. The expanded spectral range is 120–156 ppm, and only the carbon of the benzimidazole skeleton appears in this region in the benzimidazole moiety. The top spectrum (**A**) represents the liquid-state ^13^C-NMR spectrum of the tautomeric mixture of Tegoprazan. The second spectrum (**B**) from the top corresponds to the solid-state ^13^C-NMR spectrum of Polymorph A, while the third spectrum (**C**) from the top represents the solid-state ^13^C-NMR spectrum of Polymorph B. At the bottom, the spectrum (**d**) shows only the peaks corresponding to Tautomer 1, extracted from the top spectrum. Similarly, the second spectrum (**c**) from the bottom displays only the peaks corresponding to Tautomer 2, also extracted from the bottom spectrum. Spectra (**a**) and (**b**) provide expanded views of regions (**B**) and (**C**), respectively, for enhanced clarity.

The ^13^C-NMR results for each tautomer identified in Section 2.1.1 are mixed-state NMR spectra of the two tautomers; so, they were separated into separate charts to facilitate comparison with solid-state ^13^C-NMR. This was performed using the Mnova program. Their spectra were compared to the solid-state ^13^C-NMR of Polymorphs A and B, as shown in Figure 3.

Figure 3A presents the ^13^C-NMR spectrum of Tegoprazan measured in DMSO-*d6*, where all the carbon peaks of Tegoprazan are clearly observed. While Tegoprazan dissolves well in CDCl_3_, the rapid hydrogen transfer due to tautomerism makes some peaks very weak and difficult to detect. In contrast, the use of DMSO-*d6* allows for clear observation of the carbon peaks corresponding to both Tautomer 1 and Tautomer 2.

Figure 3B,C show the solid-state ^13^C-NMR spectra of Polymorphs A and B of Tegoprazan, respectively. Although the shape of the spectra differs from that of the liquid-state spectrum in Figure 3A, they appear similar overall. Characteristic peaks such as the methyl group attached to the imidazole moiety’s C2, the two methyl groups of the amide, and the carbonyl group are readily distinguishable and consistently appear as paired peaks. Other peaks also appear to follow this pairing pattern.

This observation suggests two possible scenarios: (1) the presence of both tautomers in the crystal due to tautomerism or (2) a single tautomer exhibiting conformational differences due to hydrogen bonding or intermolecular interactions, giving the appearance of two distinct molecules. The significant structural difference between Tautomer 1 and Tautomer 2 lies in the benzimidazole moiety. The chemical shift region between 125 and 156 ppm, highlighted by the red circles in the structure shown on the right side of Figure 3, corresponds to carbons observed only in the liquid-state NMR spectrum. An enlarged view of this region is shown at the bottom left of Figure 3.

Figure 3c,d display extracted spectra corresponding to Tautomer 1 and Tautomer 2, respectively, from the liquid-state spectrum in Figure 3A. In the solid-state spectra (Figure 3B,C), broader peaks compared to sharper ones suggest overlap due to closely spaced chemical shifts. The paired peaks in the spectra appear adjacent to each other. Polymorphs A and B exhibit similar patterns despite slight differences in chemical shifts.

By assigning AB to C2, CD to C4, EF to C7a, GH to C3a, and IJ to C6, the spectral assignments become consistent. However, no reasonable assignments can be made for Tautomer 2 based on the observed peaks. Thus, it is concluded that both Polymorphs A and B predominantly contain Tautomer 1. If Tautomer 2 were present in the crystal, peaks corresponding to the C7 and C7a carbons of Tautomer 2 would have been observed in the solid-state spectra.

The exclusive presence of Tautomer 1 in the crystal indicates that two distinct molecules exist in the asymmetric unit (Z′ = 2). This conclusion is consistent with the appearance of paired peaks for each carbon in the solid-state ^13^C-NMR spectra. If both Tautomer 1 and Tautomer 2 coexisted in the crystal with Z′ = 2, a total of 20 distinct carbon peaks would be observed. However, the spectra of Polymorphs A and B support the conclusion that both have the structure of Tautomer 1. This resembles the peak characteristics of the solid-state ^13^C-NMR spectrum of testosterone with an α-form polymorph (Z′ = 2) [23].

### 2.2. Structure Determination Using Laboratory X-Ray Diffraction Data

#### 2.2.1. Conformational Analysis of Tegoprazan Prior to Polymorph A and B Refinement

Using the results from the liquid- and solid-state ^13^C-NMR analysis, which indicated that Tegoprazan exists as Tautomer 1 in the crystalline state, we initiated conformational energy calculations before performing simulated annealing and Rietveld refinement for Polymorphs A and B. Tegoprazan contains seven rotatable bonds (excluding hydrogens), allowing for numerous possible conformers. Since crystallization typically occurs in the lowest-energy conformational state, theoretical calculations were performed to identify the conformer most suitable for crystallization.

**Figure 4:**
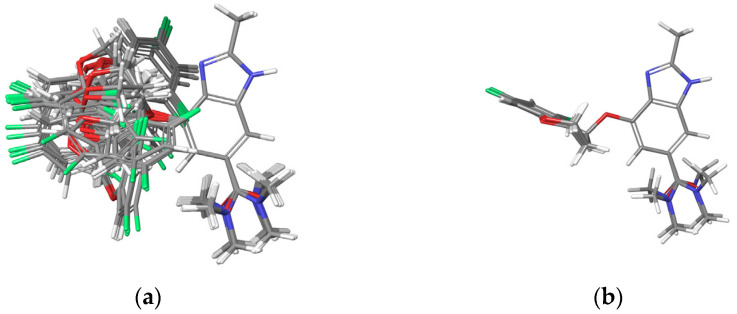
Overlay of the 44 conformers of Tegoprazan Tautomer 1 after energy minimization, using the Superimpose function in Maestro with the benzimidazole moiety fixed as the reference. (**a**) Superimposition of all 44 conformers. (**b**) Superimposition of the four lowest-energy conformers among the 44 conformers. The energy difference between the highest- and lowest-energy conformers among these four was 0.2 kcal/mol.

Initially, the Conformational Search feature of the Schrödinger software (Schrödinger Suite 2023-1) was employed, resulting in 44 conformers. These conformers were overlaid using the benzimidazole moiety as the fixed axis, as shown in Figure 4a. The force field was set to OPLS4 (Optimized Potentials for Liquid Simulations 4), with chloroform (CHCl_3_) selected as the solvent. Although vacuum conditions are often used, CHCl_3_ was chosen because Tegoprazan is known to crystallize from solvents such as dichloromethane. The method was set to Mixed torsional/Low-mode sampling, with the energy window for saving structures set to 21 kJ/mol and the maximum atom deviation cutoff set to 0.5 Å, ensuring the identification of a sufficient number of candidate conformers.

Each of the 44 conformers was subsequently geometry-optimized using the Minimize function, followed by energy calculation using the Calculation Energy feature. The same OPLS4 force field and CHCl_3_ solvent settings were applied during the energy calculations. From the resulting conformers, the four lowest-energy conformers were selected for further analysis. Their overlay is shown in Figure 4b.

In the overlay, the chromane moiety aligns closely across all four conformers, while the amide moiety exhibits variations in four distinct directions. This observation suggests that the amide region has some flexibility, with the methyl groups on the amide experiencing minimal steric hindrance, thereby allowing multiple low-energy states within an acceptable range.

The conformational search results do not account for crystal packing effects or intermolecular interactions present in the crystalline state. It is well-documented that molecular conformations often differ within crystal structures due to packing forces, as evidenced by the numerous organic compounds with Z′ values of 2 or greater reported in the literature [21]. Therefore, the conformations identified in the search do not necessarily correspond to the molecular conformations observed in the crystal structure. Nevertheless, it is reasonable to assume that these identified conformations approximate the actual molecular geometries to some extent.

#### 2.2.2. Simulated Annealing and Rietveld Refinement

Simulated annealing for crystal structure determination using powder XRD incorporates a process that randomly adjusts dihedral angles in freely rotating bonds to identify optimal conformations. If the initial structure used for this process closely resembles the actual molecular conformation within the crystal, faster convergence of the optimization is expected. The four conformers with the lowest total energy exhibit nearly identical positions for the chromane moiety, with only slight variations in the orientation of the amide group. Therefore, the choice of any of these conformers as the initial structure for simulated annealing is not expected to significantly affect the computational time required for the process. Thus, one of the four conformers was arbitrarily selected (see Supplementary Materials, Figures S30 and S31).

The powder X-ray diffraction data were collected using a RIGAKU SmartLab diffractometer operating in Bragg-Brentano geometry, configured to utilize only the Cu Kα_1_ radiation (λ = 1.540593 Å) through a monochromator. The 2*θ* range measured was from 2° to 45°, while the range used for simulated annealing and Rietveld refinement was from 5° to 35°.

Simulated annealing and Rietveld refinement were performed using the EXPO2014 program [26]. In the simulated annealing process, XRD data, the Kα_1_ wavelength from the instrument, the elemental composition, and their quantities were input into the program. Simulated annealing was selected as the structure solution method, and all options were set to automatic. The random seed was fixed at 1, and the number of runs was set to 100.

Initially, a single-molecule model was employed, but the R_wp_ (weighted profile residual factor) never dropped below 50%, indicating poor fits. Therefore, two-molecule models were used for both Polymorphs A and B.

For Polymorph A, indexing was performed using the Peak Search function in two-molecule mode. As in the single-molecule mode, 29 space groups were suggested in order of likelihood, including P2_1_, P2_1_/m, P2, P2/m, and P2_1_/n. Five space groups were selected based on agreement between the observed and calculated peak positions, and structure solutions were attempted using simulated annealing. Le Bail profile fitting yielded similar R_wp_ values for these top candidates. However, simulated annealing resulted in physically reasonable molecular packing only for the P2_1_ space group. In other space groups, either the R_wp_ remained above 30%, or, if below 30%, the resulting structures showed molecular contacts significantly shorter than van der Waals radii, or even unphysical overlaps. Consequently, the P2_1_ space group was selected and refined by the Rietveld method.

For Polymorph B, the evaluation of candidate space groups was also carried out using Le Bail profile fitting, following the same procedure used for Polymorph A. Several space groups, including P2, P2_1_, P2_1_/m, P2/m, and P2_1_/n, were tested, and the resulting R_wp_ values were comparable among the top candidates. Among these, P2 showed the best profile fit and was therefore selected for simulated annealing. However, all simulated annealing attempts for P2 produced R_wp_ values above 25%, and the resulting molecular models exhibited significant steric clashes or unphysical overlaps. Further simulated annealing trials were conducted with the other candidate space groups. Only the P2_1_ space group resulted in physically plausible molecular packing with R_wp_ values below 10%. Based on these results, P2_1_ was selected for final structure refinement.

Using the two-molecule model, simulated annealing resulted in an R_wp_ value of approximately 10%. Based on this outcome, Rietveld refinement was performed using the two-molecule structure. In the Rietveld refinement, hydrogen atom treatment was carried out with the hydrogen atom parameters constrained. A total of 68 restraints were applied, comprising 64 restraints (32 per molecule) without covalent bonds involving hydrogen and 4 angular restraints. For the hydrogen atom constraints, the atomic displacement parameter for hydrogen (ADP(H)) was set to 1.20, describing the amplitude and range of thermal vibrations in the crystal structure analysis.

The values for the restraints were determined by averaging bond lengths and angles obtained from searchable derivatives of benzimidazole and chromane in the CCDC crystal database. These values were compared to theoretical values derived from the structure optimization of the Tegoprazan molecule. Specifically, bond distances and four C–O–C angles centered on oxygen atoms were used as references.

The peak shape function was modeled using the Pearson VII function, and the March–Dollase preferred orientation correction was applied to account for crystallographic texture. For the orientation correction, various *hkl* planes (e.g., 100, 010, 001, 110, 101, 011, and 111) were systematically tested to identify the most effective direction for improving refinement accuracy. For both Polymorphs A and B, the best results were obtained using the *hkl* = 100 orientation.

**Figure 5:**
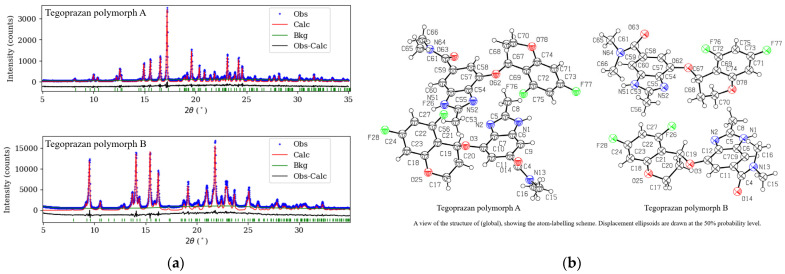
Rietveld fit to the powder X-ray diffraction data (**a**) and the ellipsoid representation of the structures of Tegoprazan Polymorphs A and B (**b**). The atomic numbering in (**b**) is arbitrarily assigned for the convenience of the structural analysis and differs from the numbering based on IUPAC nomenclature or that was assigned in NMR structural analysis.

Figure 5 shows the powder XRD pattern after Rietveld refinement, including the experimental data, background, and difference between the observed and calculated diffraction data based on the final structure, represented by a black solid line. A good agreement between the experimental and theoretical values is evident. The powder XRD data were collected over a 2θ range of 7° to 70°. In the initial stage of structure solution, simulated annealing was performed using the data up to 45°, where the major diffraction peaks are concentrated. Subsequently, Rietveld refinement was carried out using the full dataset up to 70° 2θ (see Supplementary Materials, Figure S32). To improve the refinement quality, an additional Rietveld refinement was performed using only the peaks below 35°, which yielded better agreement with the experimental pattern.

The analysis yielded R_wp_ values of 9.1% for Polymorph A and 4.9% for Polymorph B, with corresponding goodness-of-fit coefficients (χ^2^) of 1.271 and 3.791, respectively. These results indicate good agreement between the experimental data and the calculated XRD data for the converged crystal structures, supporting the reliability of the analysis. Table 1 presents the detailed crystal structures of Tegoprazan Polymorphs A and B.

#### 2.2.3. Challenges in Validating the Refined Crystal Structure via DFT-D Calculations

The accuracy of molecular crystal structures determined from powder X-ray diffraction (PXRD) data via Rietveld refinement can be quantitatively evaluated by comparing the experimentally refined geometry to a theoretically optimized structure. A commonly used metric for this purpose is the root-mean-square Cartesian displacement (RMSCD), calculated over all non-hydrogen atoms [27,28].

In a seminal study by van de Streek and Neumann [27], 215 organic crystal structures published in IUCr journals and determined from PXRD data were energy-minimized using dispersion-corrected density functional theory (DFT-D) and compared to their corresponding single-crystal structures. They found that for correctly determined structures, the RMSCD rarely exceeds 0.35 Å, thus establishing this value as a practical threshold for structure validation. Accordingly, we attempted to apply this validation approach to the two polymorphs of Tegoprazan.

This validation procedure involves subjecting the crystal structures obtained from Rietveld refinement to DFT-D-based geometry optimization, followed by a comparison of the input (experimental) and output (optimized) geometries to calculate RMSCD. Geometry optimization is typically performed in two or three stages:

1. Initial optimization of intramolecular degrees of freedom only.
2. Optimization under periodic boundary conditions (PBCs) with the unit cell held fixed.
3. Full relaxation of all degrees of freedom, including unit cell parameters.

Unlike optimizations of isolated molecules, crystal optimizations must account for intermolecular interactions such as van der Waals forces, π–π stacking, and hydrogen bonding. Even with dispersion corrections, the potential energy surface (PES) of a crystal is complex and rugged. At the early stages of optimization, the algorithm may lack sufficient information about the gradient and curvature of the PES—especially in anisotropic regions—leading to misdirected steps. This can trap the structure in an unfavorable local minimum, deviating from the experimentally refined geometry [28].

To mitigate these issues and improve convergence reliability, geometry optimization is typically staged to gradually introduce molecular flexibility—beginning with intramolecular relaxation, followed by constrained optimization under PBC and, finally, full relaxation. This approach helps reduce divergence risk and increases the likelihood of reaching a physically meaningful minimum.

While this validation method is widely adopted and highly useful, its application to the Tegoprazan polymorphs revealed substantial limitations in systems containing more than one independent molecule in the asymmetric unit (Z′ > 1). Due to repeated computational failures, we were unable to obtain reliable RMSCD values, suggesting that improved density functionals or optimization protocols may be required for such systems.

We tested commonly used DFT-D methods, including B3LYP-D3/6-31G(d), PBE-D3/cc-pVTZ(-F), M06-2X-D3/6-31G(d), ωB97X-D3/6-31G(d), and PBE0-D3/cc-pVTZ(-F), using the Jaguar (Schrödinger Inc., New York, NJ, USA, version 11.9, release 128) [29] and Quantum ESPRESSO [30,31] software packages (v.7.3.1). In most cases, geometry optimization either failed to converge within a week or crashed during vibrational frequency analysis due to memory limitations. When frequency analysis was able to be completed, it frequently revealed imaginary modes—indicating that the structure had not reached a true minimum. In the case of Quantum ESPRESSO, optimization also frequently failed due to insufficient memory. As a result, no reliable optimized structures were available for RMSCD evaluation in most cases.

For Polymorph A, we successfully obtained converged geometries using B3LYP-D3/6-31G(d) and PBE-D3/def2-TZVP. However, these structures deviated significantly from the experimentally refined model. When used as input for Rietveld refinement, they failed to reproduce the observed diffraction patterns. Moreover, simulated annealing initiated from these optimized structures reverted to the original experimental geometry, suggesting that the optimized structures did not represent energetically favorable packings. Frequency analysis further confirmed this by revealing multiple imaginary modes. Additional optimization attempts either failed to converge or produced results that were inconsistent with the experimental data (see Supplementary Materials, Figure S33a–d).

For Polymorph B, optimization with PBE-D3/6-31G(d) did converge, but the subsequent frequency analysis failed due to memory limitations. The resulting RMSCD value was approximately 1.10 Å—far exceeding the 0.35 Å threshold. Although three of the four independent molecules retained their original geometries, one showed significant deviation, and the simulated XRD pattern from this model diverged considerably from the experimental data (Figure S33e).

These observations suggest that DFT-D-based optimization may not be appropriate for systems containing multiple conformers with high conformational flexibility. While some may attribute convergence issues to the quality of the initial structure, in cases where conformers differ substantially and have large energy gaps, such problems are likely inherent to the system rather than to the starting geometry alone.

Geometry optimization becomes significantly more difficult for structures with Z′ > 1. In Z′ = 1 crystals, only one independent molecule exists in the asymmetric unit, and symmetry operations ensure uniform packing. Optimization tends to converge smoothly within a well-defined energy landscape. In contrast, Z′ = 2 crystals contain two independent molecules, each potentially with distinct conformational energies, leading to the following:

1. Increased degrees of freedom: Each atom in both molecules can move independently, resulting in over twice the number of degrees of freedom compared to Z′ = 1. This increases the likelihood of encountering multiple local minima and misaligned optimization paths.
2. Asymmetric convergence: When conformers differ in energy, one may undergo extensive rearrangement, while the other remains relatively unchanged. While total energy may appear to converge, the molecular geometries may not reflect true minimum-energy configurations. This discrepancy is sometimes evident in final total energies that are higher than intermediate values—suggesting trapping in a suboptimal state. Additionally, optimization often pushes the system toward a single averaged conformation, thereby altering the space group symmetry, which occurred in our case, where final structures adopted space group P1. However, these geometries produced poor Rietveld refinements and failed to reproduce experimental XRD patterns.
3. Interpretation of convergence: Even if optimization converges numerically, the resulting structure may not correspond to the global minimum. Frequency analysis is essential to confirm a true minimum, but for systems with large atoms or complex packing (Z′ > 1, co-crystals, hydrates), this step often fails or crashes due to memory limits. Running multiple optimizations from perturbed initial geometries is also computationally impractical.

Taken together, these challenges indicate that applying the validation criterion proposed by van de Streek and Neumann—which requires a direct comparison between optimized and experimental geometries—is not always feasible for Z′ > 1 systems. While it may remain useful for simpler structures, the method becomes increasingly unreliable in systems with large conformational flexibility or multiple non-equivalent molecules. Moreover, DFT-D calculations are conducted at 0 K in a vacuum and do not account for enthalpic or entropic effects present under ambient experimental conditions. This limitation further complicates the comparison between optimized and experimentally refined structures.

#### 2.2.4. Stability Comparison of the Two Polymorphs

The relative stability of the two Tegoprazan polymorphs was assessed by comparing their total energies, which were calculated via DFT-D using refined crystal structures. The validity of these calculations hinges on two criteria: successful geometry optimization and the absence of imaginary (negative) vibrational frequencies. The presence of negative frequencies indicates a saddle point on the potential energy surface and suggests that the structure has not reached a true energy minimum. In such cases, the optimized geometry may not represent a real structure, making any comparison of stability meaningless.

While comparing total energies offers a straightforward means to assess polymorphic stability, another commonly used approach involves estimating the lattice energy differences between polymorphs. In the absence of experimental sublimation data, the lattice energy *E_latt_* can be approximated using Equation (1) [32]:

(1) $$E_{latt}=\frac{E_{c}}{Z}-E_{g},$$

where *E_c_* is the total energy of the crystal, *Z* is the number of molecules in the unit cell, and *E_g_* is the energy of the isolated molecule. This definition is valid primarily for single-component molecular crystals.

If the isolated molecule energy *E_g_* is defined as that of the most stable conformer in the gas phase and if the two polymorphs differ only in terms of molecular conformation and packing, then, *E_g_* can be considered a constant for both forms. In this case, a direct comparison of the total crystal energies (*E_c_*) without computing *E_g_* would be sufficient to assess their relative stability.

Alternatively, if *E_g_* is defined as the energy of the conformer in its crystal geometry but is isolated from the lattice, one would need to compute the gas-phase energy of each conformer as it appears in the respective polymorph. However, this method assumes that each polymorph contains only one unique conformer. If multiple conformers are present (e.g., in Z′ > 1 systems), the energy differences between conformers complicate the estimation, and averaging may not accurately reflect their contribution to the lattice energy. This introduces uncertainty into the calculation and interpretation of relative stability.

As described in the previous section, geometry optimization and vibrational frequency analyses could not be successfully completed for the Tegoprazan polymorphs. Therefore, we adopted a single-point energy approach under the assumption that the experimentally determined crystal structures via Rietveld refinement are representative of the true solid-state forms. Total energies were calculated under consistent computational conditions, and the relative energy difference between the polymorphs was used to infer their relative stabilities. These computational results were then compared to experimental data, including solubility values, van’t Hoff-derived enthalpies of the solution, and DSC profiles, to evaluate whether the DFT-D-based energy ranking aligns with experimental thermodynamic stability.

Table 2 presents the total energy differences between the two polymorphs in various solvent environments. The values are expressed as the difference in the total energy of Polymorph B relative to Polymorph A, with the gas-phase (vacuum-state) energy difference serving as the baseline. A negative value indicates that Polymorph A is more stable, while a positive value indicates that Polymorph B is more stable.

Using the B3LYP-D3/6-31(d) method, the gas-phase energy difference was +16.35 kJ/mol, suggesting that Polymorph B is more stable in a vacuum. However, calculations using the PBE-D3/cc-pVTZ(-f) functional yielded a gas-phase energy difference of –7.84 kJ/mol, favoring Polymorph A. This discrepancy is likely due to the improved treatment of long-range intermolecular interactions, such as van der Waals forces, hydrogen bonding, and π–π stacking, in the PBE-D3/cc-pVTZ(-f) method. These interactions are particularly relevant in crystal lattices, making the PBE-D3/cc-pVTZ(-f) results more physically representative in this context.

These findings are consistent with previous reports in which B3LYP-D3 has shown less favorable performance compared to other density functionals, such as PBE-D3, particularly in complex DFT-D calculations involving large biological systems, including enzymes [33]. In addition, several benchmark studies related to lattice energy calculations have recommended the use of PBE-D3 in combination with dispersion-inclusive basis sets, such as cc-pVTZ(-f), as this approach has been shown to yield results in closer agreement with experimental values [34,35,36]. Taken together, these observations support the notion that calculations using the PBE-D3/cc-pVTZ(-f) method more accurately reflect real-world molecular interactions. Therefore, even under vacuum conditions, Polymorph A is inferred to be the more stable form.

To further investigate solvent effects on polymorph stability, solvent-phase total energy differences [Δ*E*^L^_A-B_ (solvent)] were compared to gas-phase energy differences [Δ*E*^G^_A-B_ (gas)] using the following expression: (Δ*E*^L^_A-B_)_solvent − (Δ*E*^G^_A-B_)_gas.

This calculation was performed for five solvents: water, DMSO, methanol, acetone, and dichloromethane. In all cases, both B3LYP-D3/6-31(d) and PBE-D3/cc-pVTZ(-f) yielded consistent negative energy differences with similar values, indicating that Polymorph A is more stable than Polymorph B across all solvent conditions. Furthermore, the stability advantage of Polymorph A increased with solvent polarity.

Solubility (*S*) measurements were conducted in water using the CheqSol system at five temperatures: 15 °C, 25 °C, 35 °C, 45 °C, and 55 °C. However, due to the hydrolytic instability of Tegoprazan at elevated temperatures and the pH-dependent nature of the CheqSol assay, reliable measurements could only be obtained at 15 °C and 25 °C. Based on the equilibrium solubility values at these two temperatures, the enthalpy of solution (Δ*H*_sol_) was estimated for each polymorph using van’t Hoff plots of ln(*S*) versus 1/T.

The equilibrium concentrations for Polymorph A were 1.246 mM (15 °C) and 1.438 mM (25 °C), while those for Polymorph B were 1.476 mM (15 °C) and 2.118 mM (25 °C). The corresponding Δ*H*_sol_ values were 143.9 J/mol for Polymorph A and 380.5 J/mol for Polymorph B.

DSC analysis further supported these findings. Polymorph A exhibited a strong endothermic peak near 227 °C, while Polymorph B showed a similar peak at around 167 °C (see Supplementary Materials, Figure S34). These results collectively indicate that Polymorph A is the more stable form.

The excellent agreement between the experimental data (solubility, Δ*H*_sol_, and DSC) and theoretical DFT-D calculations strongly supports the validity of using total energy computations to assess polymorph stability in Tegoprazan. This alignment supports the use of refined Rietveld structures and single-point energy calculations as a meaningful method for evaluating relative polymorphic stability when full geometry optimization is not feasible.

### 2.3. Crystallographic Structure and Supramolecular Features of Tegoprazan Polymorphs A and B

#### 2.3.1. Crystallographic Structure of Tegoprazan Polymorphs

Tautomerism of Tegoprazan was investigated through liquid-state NMR, and the resulting data, along with solid-state ^13^C-NMR spectra of Polymorphs A and B, confirmed that the Tegoprazan molecules in both polymorphs exist as Tautomer 1. Structural optimization was performed to generate initial structures, and the powder XRD data for the two polymorphs were analyzed using simulated annealing and Rietveld refinement, leading to the elucidation of their structures, as described above.

The crystal data for Polymorphs A and B of C_20_H_19_F_2_N_3_O_3_ (M = 387.38 g/mol) are as follows: both polymorphs are monoclinic, with space group P2_1_ (no. 4) and Z = 4, and were measured at 295 K. A total of 3001 reflections were collected (5.0° ≤ 2*Θ* ≤ 35.0°). The unit cell parameters for Polymorph A are a = 9.7638(5) Å, b = 21.5210(12) Å, c = 9.3267(5) Å, and β = 100.0857(16)°. For Polymorph B, the parameters are a = 22.4071(18) Å, b = 8.9485(7) Å, c = 9.6439(8) Å, and β = 97.3652(15)°. The volume for Polymorph A is V = 1929.50(18) Å^3^, and for Polymorph B, V = 1917.8(3) Å^3^. Both polymorphs exhibit similar physical properties, with μ(CuKα) = 0.878 and 0.875 mm^−1^, and calculated densities of Dcalc = 1.334 and 1.329 g/cm^3^, respectively. The R-factors for Polymorph A were R_p_ = 0.069 and R_wp_ = 0.091, and for Polymorph B, R_p_ = 0.037 and R_wp_ = 0.049.

**Figure 6:**
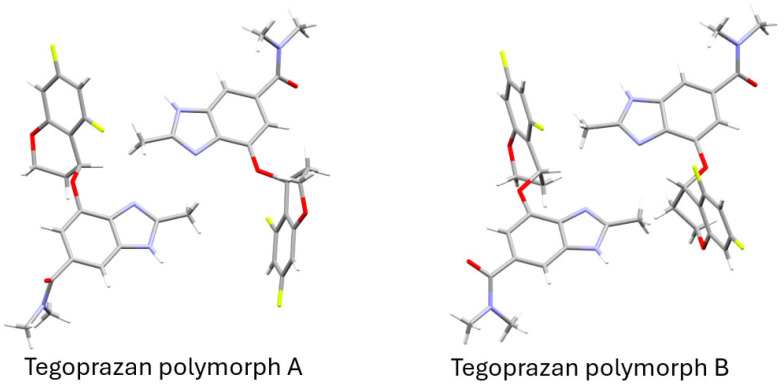
Structural representations of Tegoprazan Polymorphs A and B determined through simulated annealing and Rietveld refinement.

Figure 6 presents a simplified Capped Stick representation of the ellipsoid model shown in Figure 5b, highlighting the molecular framework with hydrogen atoms for improved clarity. In Polymorph A, one of the molecules closely resembles the initial structure used to begin the simulated annealing process, while the other molecule adopts a conformer with a total energy that is approximately 4.5 kcal/mol higher. In Polymorph B, the two molecules are nearly identical, differing only in the orientation of the amide group. As described earlier, a change in the orientation of the amide group alone has a minimal impact on the total energy. Both polymorphs are energetically less favorable than their initial structures, with Polymorph B showing an energy increase of approximately 4.5 kcal/mol. This suggests that Polymorph B is structurally less stable compared to Polymorph A. Nevertheless, it is worth noting that the packing during crystallization, as well as intermolecular interactions such as hydrogen bonding and π-π interactions, may contribute to additional stabilization. These factors suggest that the overall stability of the structure observed in the refinement results may be better understood in the context of the entire crystal rather than individual molecules in isolation.

It was determined that both of these polymorphs adopt a Z′ = 2 configuration, where two distinct molecules, rather than a single one, occupy the asymmetric unit.

#### 2.3.2. Supramolecular Features of Tegoprazan Polymorph A

**Figure 7:**
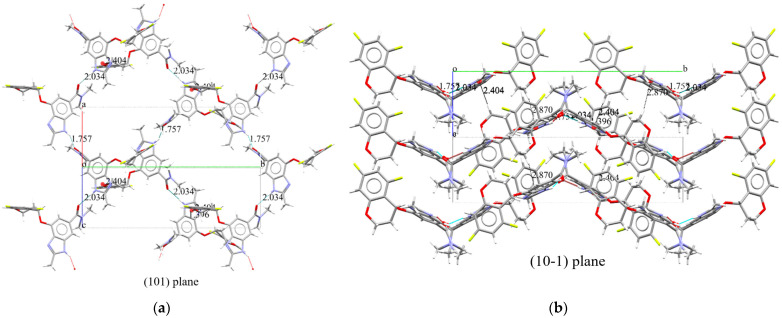
Supramolecular features of Tegoprazan Polymorph A, highlighting hydrogen bonding and π-H interactions contributing to the crystal packing stability. Hydrogen-bonding network in Tegoprazan Polymorph A as determined by simulated annealing and Rietveld refinement: A projection onto the (101) plane (**a**) reveals alternating conformers connected by hydrogen bonds with distances of 1.757 Å and 2.034 Å in the indicated direction, forming three distinct strands with no bonding in the b-direction (Table 3). A projection onto the (10-1) plane (**b**) illustrates π-H interactions stabilizing interlayer arrangements between adjacent layers. The centroid of the five-membered imidazole ring in the benzimidazole moiety is marked with a filled red circle.

Based on the crystal structure elucidated above, we can now consider the state in which a single molecule crystallizes. Focusing on the hydrogen atoms of the NH groups that are within effective hydrogen bonding distances, it can be observed that the carbonyl oxygen of the amide group interacts with these hydrogen atoms, as shown in Figure 7. In the projection of the structure onto the (101) plane (Figure 7a), alternating conformers are linked in a zigzag pattern by hydrogen bonds with distances of 1.757 Å and 2.034 Å in the direction indicated by the arrows. The figure reveals three distinct “strands,” which do not appear to interact with each other.

In the projection onto the (10-1) plane shown in Figure 7b, three layers can be observed along the horizontal direction. One of the two hydrogens on the methylene group adjacent to the oxygen of the chromane moiety forms two π-H interactions with the centroid of the imidazole ring of the benzimidazole moiety, with distances of 2.404 Å and 2.870 Å (Table 3). These interactions are believed to contribute to the stabilization of the upper and lower layers. Additionally, although the three “strands” in Figure 7a appear to be on the same plane, it is evident that the middle strand corresponds to either the upper or lower layer, as the structure suggests.

This structural arrangement highlights the role of hydrogen bonding and π-H interactions in stabilizing the overall crystal packing.

#### 2.3.3. Supramolecular Features of Tegoprazan Polymorph B

Polymorph B, as shown in Figure 6, consists of two conformers that differ solely due to the rotational changes in the carbon–carbon bond between the carbonyl carbon of the amide group and the benzimidazole moiety. This conformational difference is a result of the rotation around this bond.

**Figure 8:**
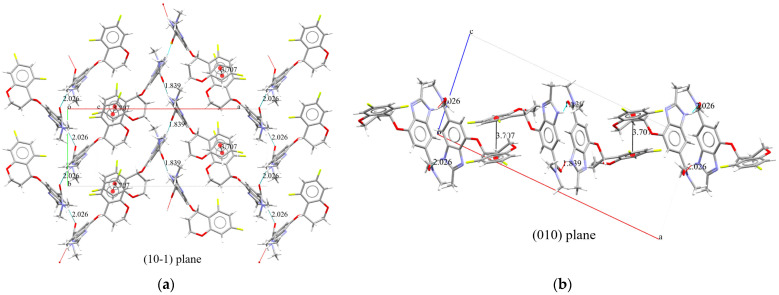
Supramolecular features of Tegoprazan Polymorph B, highlighting hydrogen bonding and π-π interactions contributing to the crystal packing stability. Hydrogen bonding and π-π interactions in Tegoprazan Polymorph B as determined by simulated annealing and Rietveld refinement: A projection onto the (10-1) plane (**a**) shows identical conformers linked by hydrogen bonds with distances of 1.839 Å and 2.026 Å in the indicated direction, forming three strands. The benzene rings of the Chromane moiety are stabilized by interactions at a centroid–centroid distance of 3.703 Å, slightly offsetting and contributing to bonding along the a-direction. A projection onto the (010) plane (**b**) reveals vertically aligned benzene rings positioned at interaction-favorable distances, suggesting stabilization through π-π interactions. The centroid of the benzene ring in the chromane moiety is marked with a filled red circle.

The crystallization behavior of Polymorph B of Tegoprazan, obtained through simulated annealing and Rietveld refinement, was observed from the projections of two planes, (010) and (10-1) (Figure 8). In the projection of the (10-1) plane (Figure 8b), hydrogen bonding interactions were confirmed between the NH-group hydrogen and the carbonyl oxygen of the amide group, with bond distances of 1.839 Å and 2.026 Å, similar to those observed in Polymorph A. Upon further inspection, it was found that identical conformers exhibit the same hydrogen bonding distances. The central strand has a distance of 1.839 Å, while the two outer strands have hydrogen bonding distances of 2.026 Å (Table 4). Additionally, although the conformers on the sides are identical, the central strand corresponds to a different conformer.

Furthermore, the fluorine-substituted benzene ring of the chromane moiety is positioned almost parallel to and overlapping with the adjacent benzene ring of the chromane moiety. The distance between the centers of these benzene rings, as seen in the projection on the (010) plane, as shown in Figure 8a, is 3.707 Å, which is within the range that allows for π-π interactions. This interaction is believed to contribute to the stabilization of the crystallization process as it involves interactions between adjacent strands. The π-π interaction is characterized by an angle of 6.023°, a centroid-to-centroid distance of 3.707 Å, and a slip distance of 0.914 Å. These interactions are anticipated to play a role in stabilizing the crystal structure.

This structural arrangement highlights the role of hydrogen bonding and π-π interactions in stabilizing the overall crystal packing.

### 2.4. Study of the Crystal Structures of Tegoprazan: Insights into Polymorphs A and B

While single-crystal XRD experiments provide definitive structural elucidation, many pharmaceutical compounds, including Tegoprazan, pose significant challenges in forming suitable single crystals. Moreover, accessing synchrotron radiation facilities can be cost-prohibitive and logistically complex. This study utilized laboratory-scale XRD instruments combined with cost-effective Rietveld refinement programs to achieve accurate structural determination.

Tautomerism in Tegoprazan was predicted based on its molecular structure and further investigated using liquid-state NMR spectroscopy. In CDCl_3_, rapid tautomerization resulted in indistinguishable peaks, whereas in DMSO-*d6*, distinct peaks corresponding to individual tautomers were observed. These findings are critical as tautomerism can influence formulation, pharmacological properties, and stability. For both Polymorphs A and B, solid-state ^13^C-NMR confirmed the presence of only Tautomer 1 within the crystal lattice.

Using Tautomer 1 as the initial structure, iterative simulated annealing and Rietveld refinement determined that both Polymorphs A and B crystallize in the monoclinic crystal system with the space group P2_1_ and Z = 4. The unit cell parameters of Polymorphs A and B are summarized in Table 1.

Supramolecular analysis revealed distinct hydrogen-bonding networks and interactions. Polymorph A forms hydrogen-bonded chains with alternating conformers stabilized by π–H interactions, while Polymorph B displays unique strands linked by hydrogen bonds and stabilized by π–π interactions between chromane moieties. Polymorph B’s conformer was found to have an energy difference of approximately 4.5 kcal/mol when compared to the lowest-energy conformer detected in conformational search studies, which corresponds to one of the conformers in Polymorph A. Despite this energy difference, the higher-energy conformer persists within Polymorph B, demonstrating the critical role of stabilizing interactions such as hydrogen bonding and π–π interactions. These findings highlight the intricate balance of forces underlying the stability of Tegoprazan polymorphs and their relevance to pharmaceutical applications.

## 3. Materials and Methods

Tegoprazan Polymorphs A and B were sourced from HK inno.N Corporation (Seoul, South Korea) and Anhui Haoyuan Pharmaceutical Co., Ltd. (Shanghai, China), respectively, and used without further modification. The polymorph samples were confirmed using XRD 2*θ* values along with ^1^H and ^13^C-NMR data.

The ^1^H, ^19^F, and ^13^C NMR spectra of crystalline forms A and B were recorded using a JNM-ECX400II spectrometer (JEOL Ltd., Tokyo, Japan) (^1^H: 400 MHz, ^13^C: 100 Hz, and ^19^F: 376 MHz) at room temperature (25 °C), with DMSO-*d6* as the solvent and tetramethylsilane (SiMe_4_) as the internal reference. The ^19^F NMR Reference Standard was trifluoroacetic acid (CF_3_COOH), which was set to −76.55 ppm. The liquid- and solid-state NMR measurement conditions are shown in Table S9. The solid-state ^13^C NMR spectra of phases A and B were acquired using cross-polarization magic-angle spinning (^13^C CP/MAS) on a Bruker AVANCE III HD 400 spectrometer (Karlsruhe, Germany). The spectra were recorded with a 4 mm zirconia rotor at a spinning speed of 12 kHz and a frequency of 100.6 MHz. The acquisition time was 37.7 milliseconds (ms), with a relaxation delay of 3 s (s), contact time of 2 ms, and 512 scans. The adamantane signal (δ = 38.48 ppm) was used as an external reference. NMR data were processed using MestReNova software (version 14.2.1-27684; Mestrelab Research, 2021).

X-ray diffraction measurements of all samples were conducted using a RIGAKU SmartLab Bragg-Brentano diffractometer in powder X-ray diffraction mode. Data were collected at 295 K in continuous 2*θ*-*θ* scan mode, with a 2*θ* range of 5°–35°. The scan rate was 1°/min, up to 20°. Single-wavelength Cu Kα_1_ radiation (λ = 1.540493 Å) was employed, with a voltage of 40 kV and a current of 50 mA, utilizing the D1 detector for signal detection.

Simulated annealing and Rietveld refinement were performed using EXPO 2014 version 1.22.11. Crystal structure imaging and various calculations were conducted with Mercury 2024.3.1 (Build 428097), Olex2 v1.5-alpha, and VESTA ver. 3.90.1a.

The conformational search, geometry optimization, single-point energy calculation, and energy minimizations were performed using the Schrödinger Suite 2023-1. The OPLS4 force field was employed for energy minimizations, and geometry optimization and single-point energy calculations were conducted using the Jaguar module and Quantum ESPRESSO at the DFT (B3LYP/6-31G(d), B3LYP-D3/6-31G(d), B3LYP-D3/6-311G(d), PBE-D3/6-31G(d), PBE-D3/6-311G(d), PBE-D3/def2-TZVP, PBE-D3/def2-SVPD, PBE-D3/cc-pVTZ-F, wω97X-D3/6-31G, M06-D3/6-31G(d), etc.) level of theory. Solvent effects were considered using the PCM (Polarizable Continuum Model) with dichloromethane as the solvent.

Solubility measurements were performed using the potentiometric (pH-metric) method with the CheqSol system (T3, Sirius Analytical Instruments Ltd., Portsmouth, UK). Unlike pKa determinations, solubility assays require relatively high sample concentrations to ensure saturation across a broad pH range (typically, pH 2 to 12). In this method, the solution is intentionally brought into a supersaturated state to establish “crossing points,” from which the intrinsic (equilibrium) solubility is calculated based on the concentration of the neutral species predominating at specific pH levels. The high initial concentration of the sample promotes supersaturation, leading to precipitation at pH values corresponding to the compound’s pKa. This onset of precipitation reflects the kinetic solubility, which can be characterized by various physicochemical parameters, including the pKa value, saturated pH, sample concentration, temperature, and crystallinity. This study aimed to investigate the temperature dependence of solubility for Polymorphs A and B. Measurements were conducted at five temperatures: 15 °C, 25 °C, 35 °C, 45 °C, and 55 °C.

DSC measurements were performed using an SDT650 instrument (TA Instruments, Waters, New Castle, DE, USA). Approximately 5–10 mg of each sample was placed in an alumina cup and heated from 30 °C to 400 °C at a rate of 10 °C/min under a nitrogen atmosphere.

## 4. Conclusions

This study demonstrates a practical approach to crystal structure analysis by employing the established Structure Determination from Powder Diffraction (SDPD) method, combined with complementary spectroscopic techniques, including liquid-state and solid-state NMR. Tegoprazan, a compound known to exhibit tautomerism, was selected as a model system. Liquid-state NMR revealed the presence of multiple tautomers in solution, with solvent-specific behavior. In CDCl_3_, rapid tautomerization resulted in indistinguishable NMR peaks, while in DMSO-*d6*, distinct peaks corresponding to individual tautomers were observed. In liquid NMR, the NH proton was detected at 12.4 ppm and identified as belonging to Tautomer 2. This peak was consistently observed in both Polymorph A and Polymorph B. Solid-state ^13^C-NMR provided crucial evidence confirming the exclusive presence of Tautomer 1 within the crystal lattices of Polymorphs A and B. These findings underscore the indispensable role of NMR spectroscopy in complementing SDPD for a comprehensive understanding of tautomeric behavior in the solid state.

Using Tautomer 1 as the initial molecular model, iterative simulated annealing and Rietveld refinement with laboratory-scale XRD instrumentation enabled the successful elucidation of the crystal structures of Polymorphs A and B. Both polymorphs were found to crystallize in the monoclinic system with the space group P2_1_ and Z = 4. Polymorph A was determined to consist of one molecule in a near-ground-state conformation and another in a higher-energy conformation, approximately 4.5 kcal/mol above the ground state. In contrast, Polymorph B comprises two identical higher-energy conformers, each with an energy of approximately 4.5 kcal/mol above the ground state. These results suggest that Polymorph A represents the thermodynamically more stable crystalline form under the investigated conditions.

To further evaluate the stability and reliability of these structures, dispersion-corrected DFT (DFT-D) calculations were performed. Full geometry optimization and vibrational analyses proved difficult due to the high degrees of freedom in Z′ = 2 systems, often resulting in convergence failure or imaginary frequencies. Therefore, a single-point energy approach was adopted using the experimentally refined structures. The calculated energy differences, supported by solubility measurements, van’t Hoff enthalpies of the solution, and DSC data, consistently indicated that Polymorph A is the more stable form. This demonstrates the utility of energy-based assessments when full geometry optimization is computationally prohibitive, particularly for conformationally complex molecular crystals.

Furthermore, the supramolecular analysis revealed that Polymorph A forms hydrogen-bonded chains stabilized by π–H interactions, while Polymorph B exhibits unique hydrogen-bonded strands stabilized by π–π interactions between chromane moieties. These findings emphasize the importance of intermolecular interactions, such as hydrogen bonding and π–π stacking, in determining polymorphic packing and stability.

In conclusion, this study illustrates the value of integrating SDPD with advanced spectroscopic and computational approaches for the investigation of pharmaceutical compounds exhibiting complex behaviors such as tautomerism and polymorphism. While SDPD itself is an established technique, its combination with complementary tools enabled a comprehensive analysis of Tegoprazan’s crystal structures and stability. This integrated methodology enhances the accessibility of crystal structure determination in resource-limited settings and provides a robust framework for future exploration of molecular conformation, lattice energy, and polymorph stability in drug development.

## Figures and Tables

**Table 1:** Results of crystal structure determination of Polymorphs A and B of Tegoprazan via simulated annealing and Rietveld refinement.

Polymorph	A	B
Crystal data		
Chemical formula	C_20_H_19_F_2_N_3_O_3_	C_20_H_19_F_2_N_3_O_3_
M_r_	387.38	387.38
Crystal system, space group	Monoclinic, P2_1_	Monoclinic, P2_1_
Temperature (K)	295	295
a, b, c (Å)	9.7638 (5), 21.5210 (12), 9.3267 (5)	22.4071 (18), 8.9485 (7), 9.6439 (8)
β (°)	100.0857 (16)	97.3652 (15)
V (Å^3^)	1929.50 (18)	1917.8 (3)
Z	4	4
Radiation type	Cu Kα_1_, λ = 1.540593 Å	Cu Kα_1_, λ = 1.540593 Å
Specimen shape, size (mm)	Flat sheet, 20 × 0.2	Flat sheet, 20 × 0.2
Data collection		
Diffractometer	RIGAKU SmartLab Bragg-Brentano Diffractometer	RIGAKU SmartLab Bragg-Brentano Diffractometer
Specimen mounting	Glass plate	Glass plate
Data collection mode	Reflection	Reflection
Scan method	Continuous	Continuous
2*θ* values (°)	2*θ*_min_ = 5.00 2*θ*_max_ = 35.00 2*θ*_step_ = 0.02	2*θ*_min_ = 5.00 2*θ*_max_ = 35.00 2*θ*_step_ = 0.02
Refinement		
R-factors and goodness of fit	R_p_ = 0.069, R_wp_ = 0.091, R_exp_ = 0.081, R_Bragg_ = 0.039, χ^2^ = 1.271	R_p_ = 0.037, R_wp_ = 0.049, R_exp_ = 0.025, R_Bragg_ = 0.019, χ^2^ = 3.791
No. of parameters	254	254
No. of restraints	68	68
H-atom treatment	H-atom parameters constrained	H-atom parameters constrained

CCDC 2376144 (Polymorph A) and 2387615 (Polymorph B) contain the supplementary crystallographic data for this paper. These data can be obtained free of charge via https://www.ccdc.cam.ac.uk/structures/Search?Ccdcid=2376144&DatabaseToSearch=Published (accessed on 7 August 2024); https://www.ccdc.cam.ac.uk/structures/Search?Ccdcid=2387615&DatabaseToSearch=Published (accessed on 29 September 2024) (or from the CCDC, 12 Union Road, Cambridge CB2 1EZ, UK; Fax: +44 1223 336033; E-mail: deposit@ccdc.cam.ac.uk).

**Table 2:** Solvent-phase energy differences between Polymorphs A and B relative to the gas phase: A comparison of B3LYP-D3/6-31** and PBE-D3/cc-pVTZ(-f) (J/mol) [(solvent-phase Δ*E*^L^_A-B_) − (gas-phase Δ*E*^G^_A-B_)] ^1^.

DFT-D (Theory/Basis Set)	Water	DMSO	Methanol	Acetone	Dichloromethane
B3LYP-D3/6-31(d)	−57.7	−57.1	−56.5	−55.2	−51.0
PBE-D3/cc-pVTZ(-f)	−57.6	−57.0	−56.3	−55.0	−50.5

^1^ Δ*E*^L^_A-B_ represents the total energy difference between Polymorphs A and B in the solvent phase, whereas Δ*E*^G^_A-B_ corresponds to the total energy difference between the two polymorphs in the gas phase (vacuum state). 6-31G** includes extra functions for more flexible electron distribution, improving results for both heavy atoms and hydrogens.

**Table 3:** Hydrogen-bond geometry of Tegoprazan phase A.

D–H⋯A	D–H	H⋯A	D⋯A	D–H⋯A
N1–H46 A ⋯O63 ^i^	0.8699	2.0335	2.9025(11)	176.84
N51–H94 A ⋯O14 ^ii^	0.8699	1.7566	2.5555(10)	151.58
C70–H92 A ⋯Cg1 ^iii^	0.97	2.404	3.354(1)	166.11
C17–H40 A ⋯Cg2 ^iii^	0.97	2.870	3.820(1)	166.27

Symmetry codes: (^i^) −x + 1, y−1/2, −z + 1; (^ii^) −x + 2, y + 1/2, −z; (^iii^) 1 + x, y, z. Hydrogen-bond geometry (Å, °); Cg1 and Cg2 are the centroids of the N1-C5-N2-C7-C6 and N51-C53-N52-C54-C55 rings, respectively.

**Table 4:** Hydrogen-bond geometry of Tegoprazan phase B.

D–H⋯A	D–H	H⋯A	D⋯A	D–H⋯A
N1–H46 A ⋯O14 ^i^	0.8699	1.8388	2.6171(16)	147.93
N51–H94 A ⋯O63 ^ii^	0.8698	2.0259	2.8210(16)	151.5

Symmetry codes: (^i^) −x, y + 1/2, -z; (^ii^) −x + 1, y + 1/2, −z. Hydrogen-bond geometry (Å, °).

## Data Availability

The original contributions presented in this study are included in the article/Supplementary Material. Further inquiries can be directed to the corresponding author(s).

## Supplementary Materials

The following supporting information can be downloaded at: https://www.mdpi.com/article/10.3390/molecules30071538/s1, Table S1: Liquid NMR data of Tegoprazan Tautomer 1; Table S2: Liquid NMR chemical shifts of Tegoprazan Tautomer 2; Table S3: Liquid- and solid-state NMR measurement conditions; Table S4: SS ^13^C-NMR chemical shift of Tegoprazan phase A; Table S5: SS ^13^C-NMR chemical shift of Tegoprazan phase B; Figure S1: Tegoprazan: ^1^H-NMR—Tautomer 1 with chemical shifts; Figure S2: Tegoprazan: ^1^H-NMR—Tautomer 1 (expanded); Figure S3:Tegoprazan: ^13^C-NMR—Tautomer 1 with chemical shifts; Figure S4: Tegoprazan: ^13^C-NMR—Tautomer 1 (expanded); Figure S5: Tegoprazan: ^19^F-NMR—Tautomer 1 with chemical shifts; Figure S6: Tegoprazan: ^19^F-NMR—Tautomer 1 (expanded); Figure S7: Tegoprazan: ^1^H-^1^H COSY—Tautomer 1 (assigned); Figure S8: Tegoprazan: Overlay of ^1^H-^13^C HSQC and HMBC—Tautomer 1 (expanded); Figure S9: Tegoprazan: ^1^H-^13^C HSQC—Tautomer 1; Figure S10: Tegoprazan: ^1^H-^13^C HMBC—Tautomer 1; Figure S11: Tegoprazan: ^1^H-^1^H ROESY—Tautomer 1; Figure S12: Tegoprazan: ^19^F-^13^C HSQC—Tautomer 1; Figure S13: Tegoprazan: ^19^F-^13^C HMBC—Tautomer 1; Figure S14: Tegoprazan ^1^H-NMR—Tautomer 2 with chemical shifts; Figure S15: Tegoprazan ^1^H-NMR—Tautomer 2 (expanded); Figure S16: Tegoprazan ^13^C-NMR—Tautomer 2 with chemical shifts; Figure S17: Tegoprazan ^13^C-NMR—Tautomer 2 (expanded); Figure S18: Tegoprazan ^19^F-NMR—Tautomer 2 with chemical shifts; Figure S19: Tegoprazan ^19^F-NMR—Tautomer 2 (expanded); Figure S20: Tegoprazan ^1^H-^1^H COSY—Tautomer 2 (assigned); Figure S21: Tegoprazan: Overlay of ^1^H-^13^C HSQC and HMBC—Tautomer 2 (expanded); Figure S22: Tegoprazan: ^1^H-^13^C HSQC—Tautomer 2; Figure S23: Tegoprazan: ^1^H-^13^C HMBC—Tautomer 2; Figure S24: Tegoprazan: ^1^H-^1^H ROESY—Tautomer 2; Figure S25: Correlations between the NH proton and C7a (a), C3a (b), and C2 (c) [19]; Figure S26: Tegoprazan: ^19^F-^13^C HSQC—Tautomer 2; Figure S27: Tegoprazan: ^19^F-^13^C HMBC—Tautomer 2; Figure S28: Tegoprazan: Solid-state ^13^C-NMR—Phase A; Figure S29: Tegoprazan: Solid-state ^13^C-NMR—Phase B; Figure S30: Relative total energies (ΔE_rel_) (kcal/mol) of conformers of Tegoprazan; Figure S31: Initial structure used for determining the crystal structure of Tegoprazan Polymorph A and B; Figure S32. Initial Rietveld refinement profiles of Tegoprazan Polymorphs A and B over the 2θ range of 7° to 70°; Figure S33: Superimposed molecular structures of Tegoprazan Polymorphs A and B obtained from DFT-D geometry optimization; Table S6: Relative energies of the four most stable conformers (total energy = prime energy); Table S7: Hydrogen-bond geometry of Tegoprazan phase A; Table S8: Hydrogen-bond geometry of Tegoprazan phase B; Figure S34: DSC and XRD data of Tegoprazan Polymorph B at various time points during the stability test; Table S9: Summary of XRD characteristic peaks and normalized intensities for Tegoprazan Polymorph A; and Table S10: Summary of XRD characteristic peaks and normalized intensities for Tegoprazan Polymorph B.

## Abbreviations

The following abbreviations are used in this manuscript:

P-CAB	potassium ion-competitive acid blocker
PPIs	proton pump inhibitors
GERD	gastroesophageal reflux disease
XRD	X-ray diffraction
PXRD	powder X-ray diffraction
DSC	Differential Scanning Calorimetry
SDPD	Structure Determination from Powder Diffraction
COSY	Correlation Spectroscopy
ROESY	Rotating Frame Overhauser Enhancement Spectroscopy
HSQC	Heteronuclear Single Quantum Coherence
HMBC	Heteronuclear Multiple Bond Coherence
OPLS4	Optimized Potentials for Liquid Simulations 4
IUPAC	International Union of Pure and Applied Chemistry
ADP(H)	atomic displacement parameter for hydrogen
CCDC	Cambridge Crystallographic Data Centre
PCM	Polarizable Continuum Model
DFT-D	dispersion-corrected density functional theory
PBCs	periodic boundary conditions
RMSCD	root-mean-square Cartesian displacement
R_wp_	weighted profile residual factor
SDPD	Structure Determination from Powder Diffraction
