# Supplementary material for: Structure Determination of Tegoprazan((S)-4-((5,7-difluorochroman-4-yl)oxy)-N,N,2-trimethyl-1H-benzo[d]imidazole-6-formamide) Polymorphs A and B by Laboratory X-Ray Powder Diffraction
Source: Molecules. 2025 Mar 30;30(7):1538. doi: 10.3390/molecules30071538 (PMC11990383; doi:10.3390/molecules30071538)
Supplement: Supplementary file 1 [file molecules-30-01538-s001.zip › molecules-3467479-supplementary.pptx]

## Slide 1
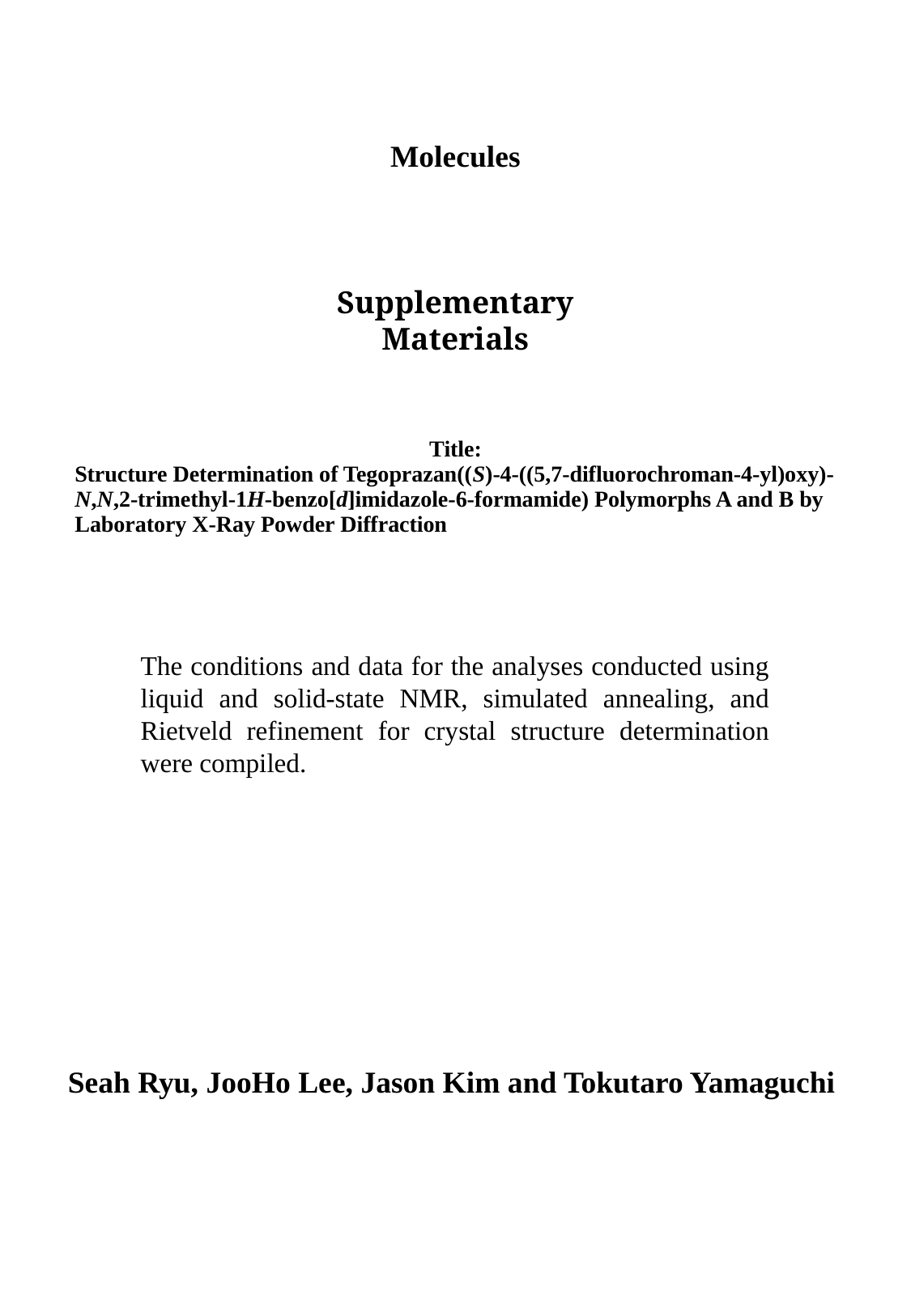

Molecules
Supplementary Materials
| Title: Structure Determination of Tegoprazan((S)-4-((5,7-difluorochroman-4-yl)oxy)-N,N,2-trimethyl-1H-benzo[d]imidazole-6-formamide) Polymorphs A and B by Laboratory X-Ray Powder Diffraction |
| --- |
The conditions and data for the analyses conducted using liquid and solid-state NMR, simulated annealing, and Rietveld refinement for crystal structure determination were compiled.
Seah Ryu, JooHo Lee, Jason Kim and Tokutaro Yamaguchi

## Slide 2
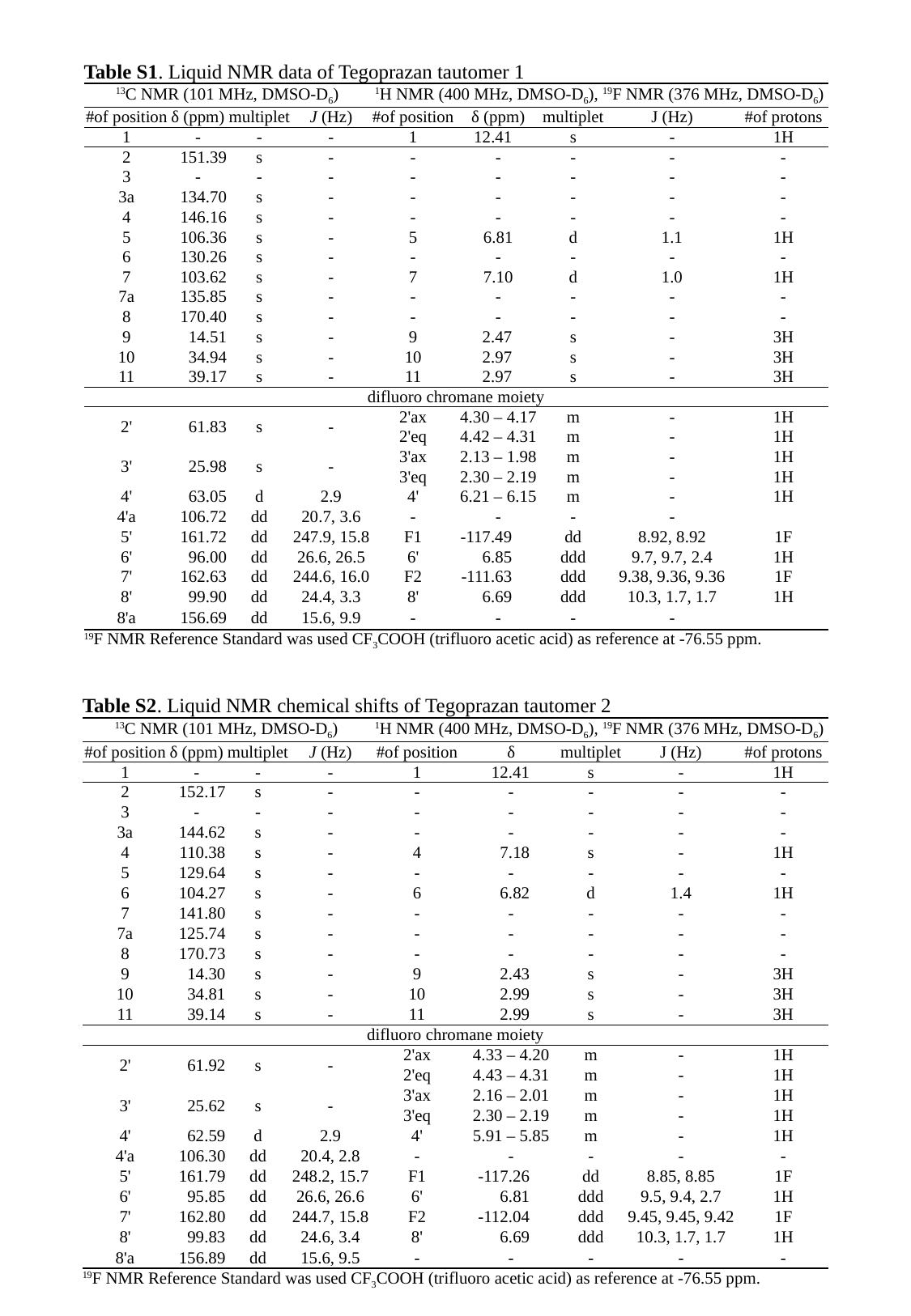

| Table S1. Liquid NMR data of Tegoprazan tautomer 1 | | | | | | | | |
| --- | --- | --- | --- | --- | --- | --- | --- | --- |
| 13C NMR (101 MHz, DMSO-D6) | | | | 1H NMR (400 MHz, DMSO-D6), 19F NMR (376 MHz, DMSO-D6) | | | | |
| #of position | δ (ppm) | multiplet | J (Hz) | #of position | δ (ppm) | multiplet | J (Hz) | #of protons |
| 1 | - | - | - | 1 | 12.41 | s | - | 1H |
| 2 | 151.39 | s | - | - | - | - | - | - |
| 3 | - | - | - | - | - | - | - | - |
| 3a | 134.70 | s | - | - | - | - | - | - |
| 4 | 146.16 | s | - | - | - | - | - | - |
| 5 | 106.36 | s | - | 5 | 6.81 | d | 1.1 | 1H |
| 6 | 130.26 | s | - | - | - | - | - | - |
| 7 | 103.62 | s | - | 7 | 7.10 | d | 1.0 | 1H |
| 7a | 135.85 | s | - | - | - | - | - | - |
| 8 | 170.40 | s | - | - | - | - | - | - |
| 9 | 14.51 | s | - | 9 | 2.47 | s | - | 3H |
| 10 | 34.94 | s | - | 10 | 2.97 | s | - | 3H |
| 11 | 39.17 | s | - | 11 | 2.97 | s | - | 3H |
| difluoro chromane moiety | | | | | | | | |
| 2' | 61.83 | s | - | 2'ax | 4.30 – 4.17 | m | - | 1H |
| | | | | 2'eq | 4.42 – 4.31 | m | - | 1H |
| 3' | 25.98 | s | - | 3'ax | 2.13 – 1.98 | m | - | 1H |
| | | | | 3'eq | 2.30 – 2.19 | m | - | 1H |
| 4' | 63.05 | d | 2.9 | 4' | 6.21 – 6.15 | m | - | 1H |
| 4'a | 106.72 | dd | 20.7, 3.6 | - | - | - | - | |
| 5' | 161.72 | dd | 247.9, 15.8 | F1 | -117.49 | dd | 8.92, 8.92 | 1F |
| 6' | 96.00 | dd | 26.6, 26.5 | 6' | 6.85 | ddd | 9.7, 9.7, 2.4 | 1H |
| 7' | 162.63 | dd | 244.6, 16.0 | F2 | -111.63 | ddd | 9.38, 9.36, 9.36 | 1F |
| 8' | 99.90 | dd | 24.4, 3.3 | 8' | 6.69 | ddd | 10.3, 1.7, 1.7 | 1H |
| 8'a | 156.69 | dd | 15.6, 9.9 | - | - | - | - | |
| 19F NMR Reference Standard was used CF3COOH (trifluoro acetic acid) as reference at -76.55 ppm. | | | | | | | | |
| Table S2. Liquid NMR chemical shifts of Tegoprazan tautomer 2 | | | | | | | | |
| --- | --- | --- | --- | --- | --- | --- | --- | --- |
| 13C NMR (101 MHz, DMSO-D6) | | | | 1H NMR (400 MHz, DMSO-D6), 19F NMR (376 MHz, DMSO-D6) | | | | |
| #of position | δ (ppm) | multiplet | J (Hz) | #of position | δ | multiplet | J (Hz) | #of protons |
| 1 | - | - | - | 1 | 12.41 | s | - | 1H |
| 2 | 152.17 | s | - | - | - | - | - | - |
| 3 | - | - | - | - | - | - | - | - |
| 3a | 144.62 | s | - | - | - | - | - | - |
| 4 | 110.38 | s | - | 4 | 7.18 | s | - | 1H |
| 5 | 129.64 | s | - | - | - | - | - | - |
| 6 | 104.27 | s | - | 6 | 6.82 | d | 1.4 | 1H |
| 7 | 141.80 | s | - | - | - | - | - | - |
| 7a | 125.74 | s | - | - | - | - | - | - |
| 8 | 170.73 | s | - | - | - | - | - | - |
| 9 | 14.30 | s | - | 9 | 2.43 | s | - | 3H |
| 10 | 34.81 | s | - | 10 | 2.99 | s | - | 3H |
| 11 | 39.14 | s | - | 11 | 2.99 | s | - | 3H |
| difluoro chromane moiety | | | | | | | | |
| 2' | 61.92 | s | - | 2'ax | 4.33 – 4.20 | m | - | 1H |
| | | | | 2'eq | 4.43 – 4.31 | m | - | 1H |
| 3' | 25.62 | s | - | 3'ax | 2.16 – 2.01 | m | - | 1H |
| | | | | 3'eq | 2.30 – 2.19 | m | - | 1H |
| 4' | 62.59 | d | 2.9 | 4' | 5.91 – 5.85 | m | - | 1H |
| 4'a | 106.30 | dd | 20.4, 2.8 | - | - | - | - | - |
| 5' | 161.79 | dd | 248.2, 15.7 | F1 | -117.26 | dd | 8.85, 8.85 | 1F |
| 6' | 95.85 | dd | 26.6, 26.6 | 6' | 6.81 | ddd | 9.5, 9.4, 2.7 | 1H |
| 7' | 162.80 | dd | 244.7, 15.8 | F2 | -112.04 | ddd | 9.45, 9.45, 9.42 | 1F |
| 8' | 99.83 | dd | 24.6, 3.4 | 8' | 6.69 | ddd | 10.3, 1.7, 1.7 | 1H |
| 8'a | 156.89 | dd | 15.6, 9.5 | - | - | - | - | - |
| 19F NMR Reference Standard was used CF3COOH (trifluoro acetic acid) as reference at -76.55 ppm. | | | | | | | | |

## Slide 3
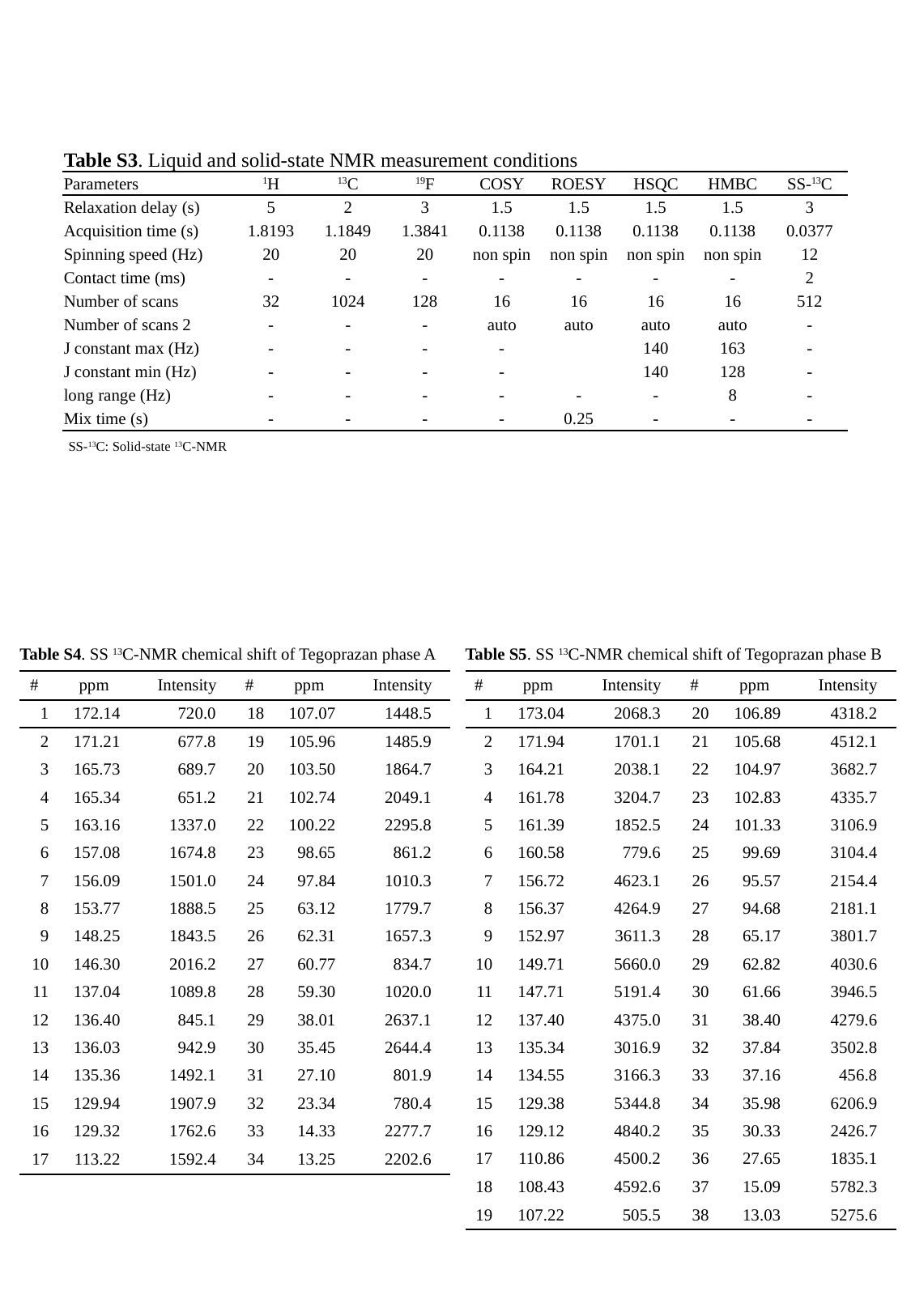

| Table S3. Liquid and solid-state NMR measurement conditions | | | | | | | | |
| --- | --- | --- | --- | --- | --- | --- | --- | --- |
| Parameters | 1H | 13C | 19F | COSY | ROESY | HSQC | HMBC | SS-13C |
| Relaxation delay (s) | 5 | 2 | 3 | 1.5 | 1.5 | 1.5 | 1.5 | 3 |
| Acquisition time (s) | 1.8193 | 1.1849 | 1.3841 | 0.1138 | 0.1138 | 0.1138 | 0.1138 | 0.0377 |
| Spinning speed (Hz) | 20 | 20 | 20 | non spin | non spin | non spin | non spin | 12 |
| Contact time (ms) | - | - | - | - | - | - | - | 2 |
| Number of scans | 32 | 1024 | 128 | 16 | 16 | 16 | 16 | 512 |
| Number of scans 2 | - | - | - | auto | auto | auto | auto | - |
| J constant max (Hz) | - | - | - | - | | 140 | 163 | - |
| J constant min (Hz) | - | - | - | - | | 140 | 128 | - |
| long range (Hz) | - | - | - | - | - | - | 8 | - |
| Mix time (s) | - | - | - | - | 0.25 | - | - | - |
SS-13C: Solid-state 13C-NMR
| Table S4. SS 13C-NMR chemical shift of Tegoprazan phase A | | | | | |
| --- | --- | --- | --- | --- | --- |
| # | ppm | Intensity | # | ppm | Intensity |
| 1 | 172.14 | 720.0 | 18 | 107.07 | 1448.5 |
| 2 | 171.21 | 677.8 | 19 | 105.96 | 1485.9 |
| 3 | 165.73 | 689.7 | 20 | 103.50 | 1864.7 |
| 4 | 165.34 | 651.2 | 21 | 102.74 | 2049.1 |
| 5 | 163.16 | 1337.0 | 22 | 100.22 | 2295.8 |
| 6 | 157.08 | 1674.8 | 23 | 98.65 | 861.2 |
| 7 | 156.09 | 1501.0 | 24 | 97.84 | 1010.3 |
| 8 | 153.77 | 1888.5 | 25 | 63.12 | 1779.7 |
| 9 | 148.25 | 1843.5 | 26 | 62.31 | 1657.3 |
| 10 | 146.30 | 2016.2 | 27 | 60.77 | 834.7 |
| 11 | 137.04 | 1089.8 | 28 | 59.30 | 1020.0 |
| 12 | 136.40 | 845.1 | 29 | 38.01 | 2637.1 |
| 13 | 136.03 | 942.9 | 30 | 35.45 | 2644.4 |
| 14 | 135.36 | 1492.1 | 31 | 27.10 | 801.9 |
| 15 | 129.94 | 1907.9 | 32 | 23.34 | 780.4 |
| 16 | 129.32 | 1762.6 | 33 | 14.33 | 2277.7 |
| 17 | 113.22 | 1592.4 | 34 | 13.25 | 2202.6 |
| Table S5. SS 13C-NMR chemical shift of Tegoprazan phase B | | | | | |
| --- | --- | --- | --- | --- | --- |
| # | ppm | Intensity | # | ppm | Intensity |
| 1 | 173.04 | 2068.3 | 20 | 106.89 | 4318.2 |
| 2 | 171.94 | 1701.1 | 21 | 105.68 | 4512.1 |
| 3 | 164.21 | 2038.1 | 22 | 104.97 | 3682.7 |
| 4 | 161.78 | 3204.7 | 23 | 102.83 | 4335.7 |
| 5 | 161.39 | 1852.5 | 24 | 101.33 | 3106.9 |
| 6 | 160.58 | 779.6 | 25 | 99.69 | 3104.4 |
| 7 | 156.72 | 4623.1 | 26 | 95.57 | 2154.4 |
| 8 | 156.37 | 4264.9 | 27 | 94.68 | 2181.1 |
| 9 | 152.97 | 3611.3 | 28 | 65.17 | 3801.7 |
| 10 | 149.71 | 5660.0 | 29 | 62.82 | 4030.6 |
| 11 | 147.71 | 5191.4 | 30 | 61.66 | 3946.5 |
| 12 | 137.40 | 4375.0 | 31 | 38.40 | 4279.6 |
| 13 | 135.34 | 3016.9 | 32 | 37.84 | 3502.8 |
| 14 | 134.55 | 3166.3 | 33 | 37.16 | 456.8 |
| 15 | 129.38 | 5344.8 | 34 | 35.98 | 6206.9 |
| 16 | 129.12 | 4840.2 | 35 | 30.33 | 2426.7 |
| 17 | 110.86 | 4500.2 | 36 | 27.65 | 1835.1 |
| 18 | 108.43 | 4592.6 | 37 | 15.09 | 5782.3 |
| 19 | 107.22 | 505.5 | 38 | 13.03 | 5275.6 |

## Slide 4
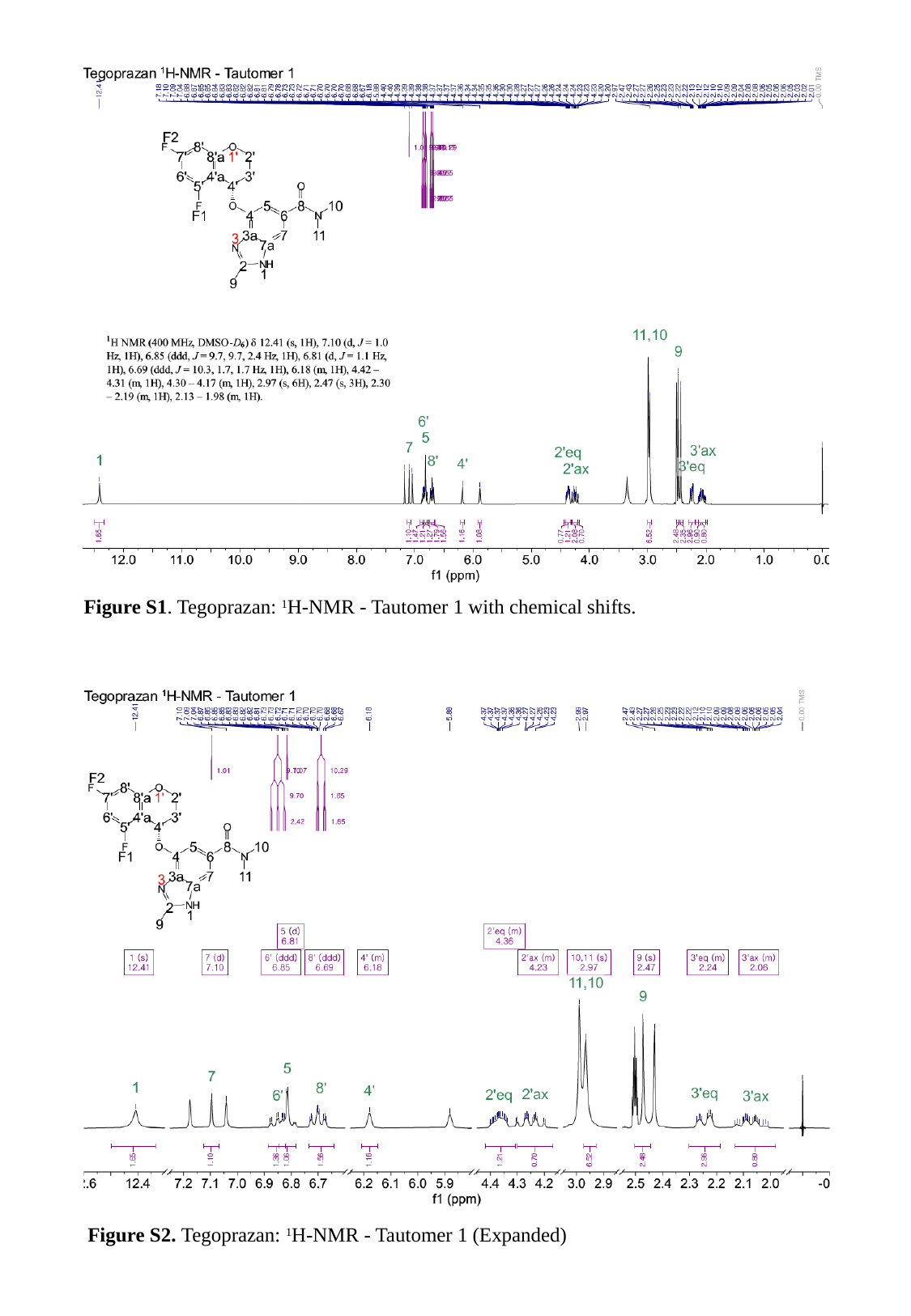

Figure S1. Tegoprazan: 1H-NMR - Tautomer 1 with chemical shifts.
Figure S2. Tegoprazan: 1H-NMR - Tautomer 1 (Expanded)

## Slide 5
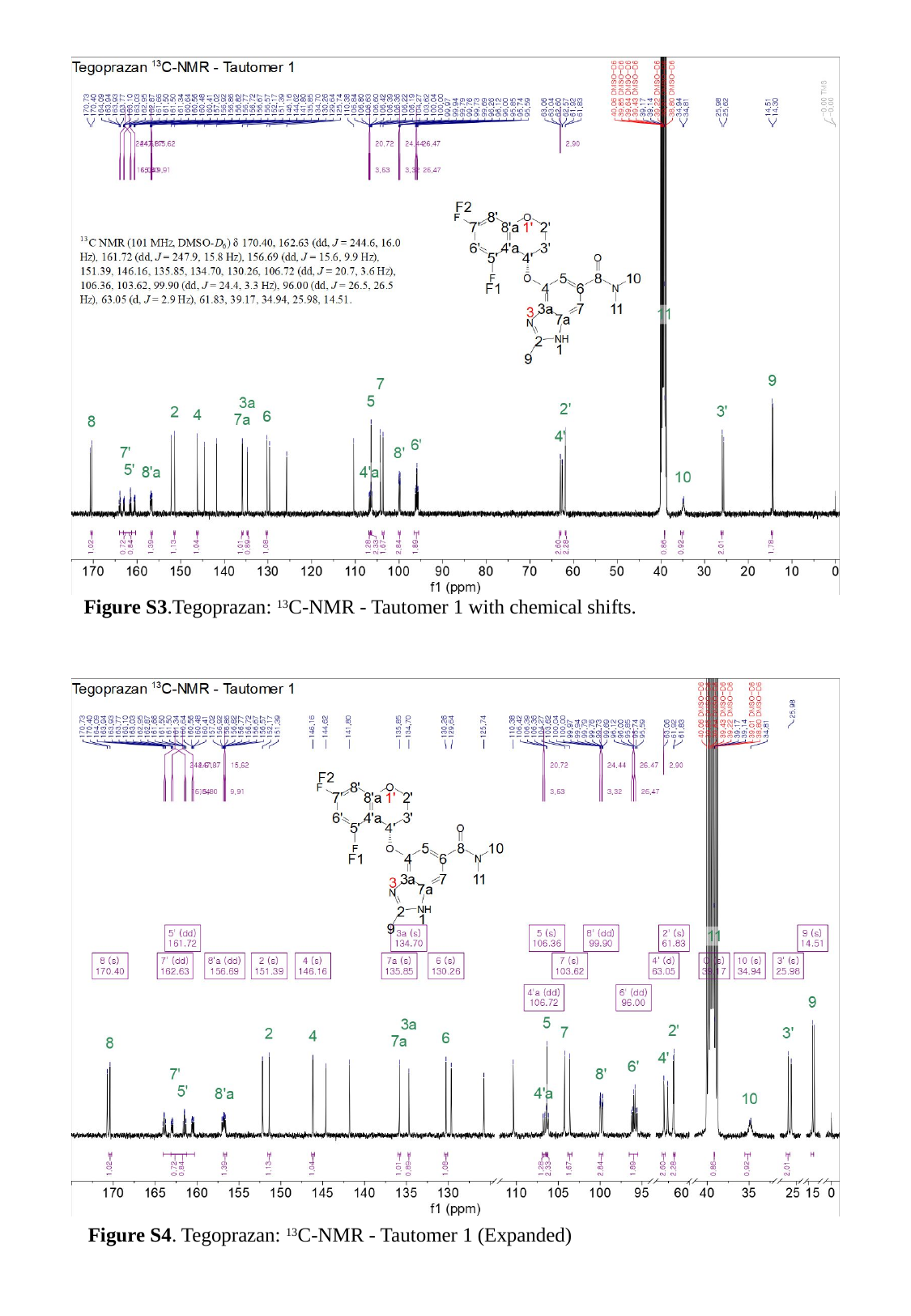

Figure S3.Tegoprazan: 13C-NMR - Tautomer 1 with chemical shifts.
Figure S4. Tegoprazan: 13C-NMR - Tautomer 1 (Expanded)

## Slide 6
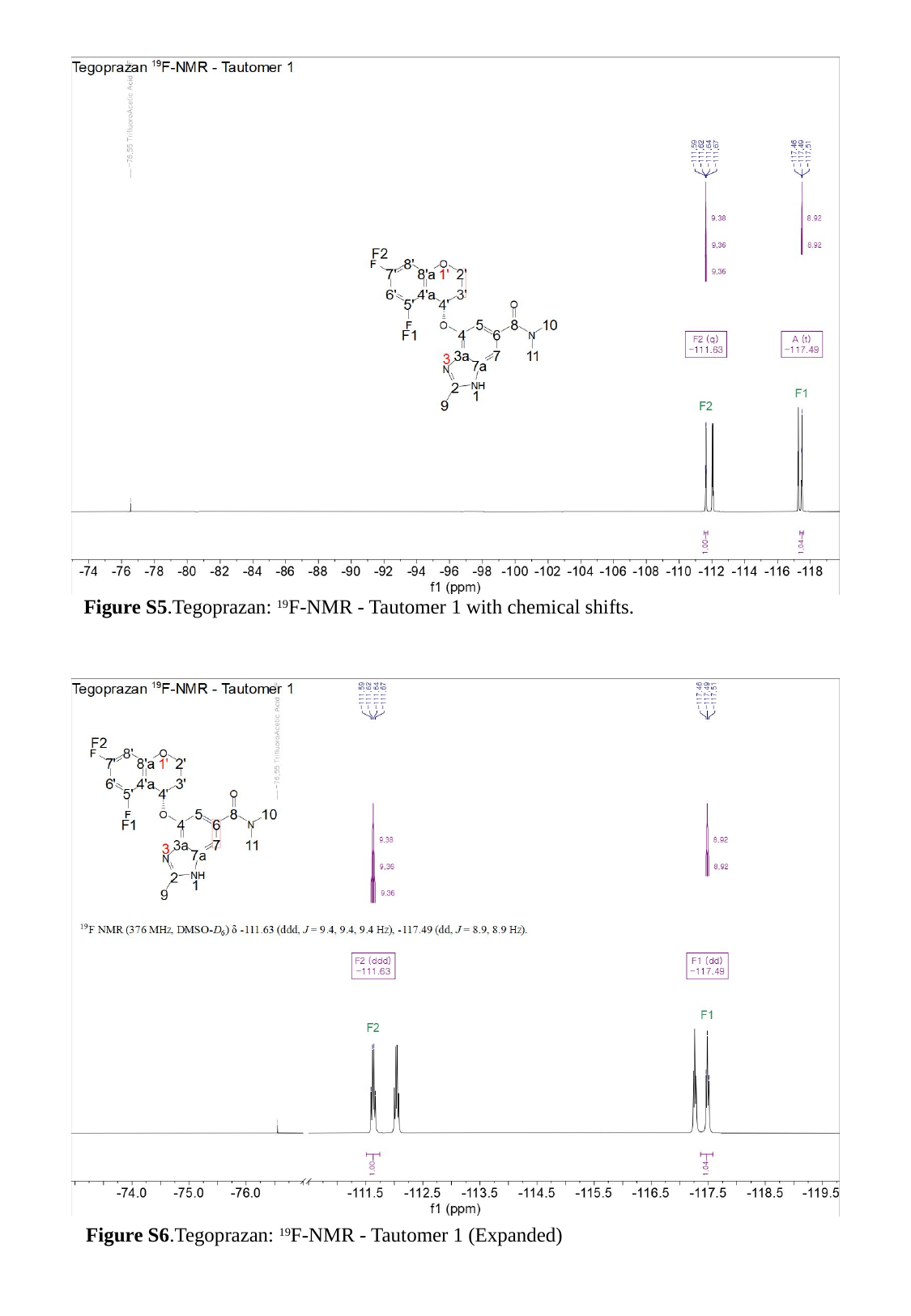

Figure S5.Tegoprazan: 19F-NMR - Tautomer 1 with chemical shifts.
Figure S6.Tegoprazan: 19F-NMR - Tautomer 1 (Expanded)

## Slide 7
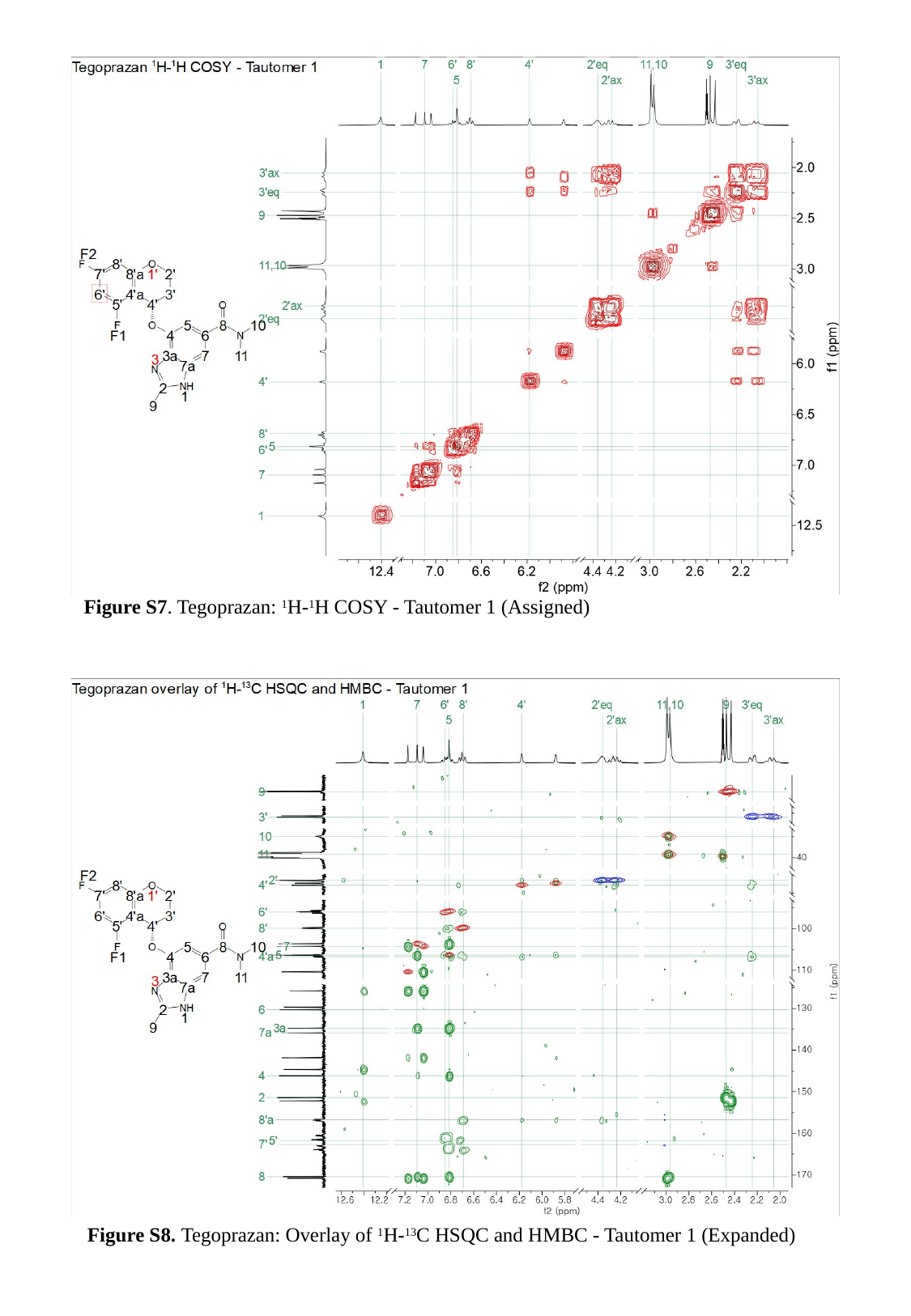

Figure S7. Tegoprazan: 1H-1H COSY - Tautomer 1 (Assigned)
Figure S8. Tegoprazan: Overlay of 1H-13C HSQC and HMBC - Tautomer 1 (Expanded)

## Slide 8
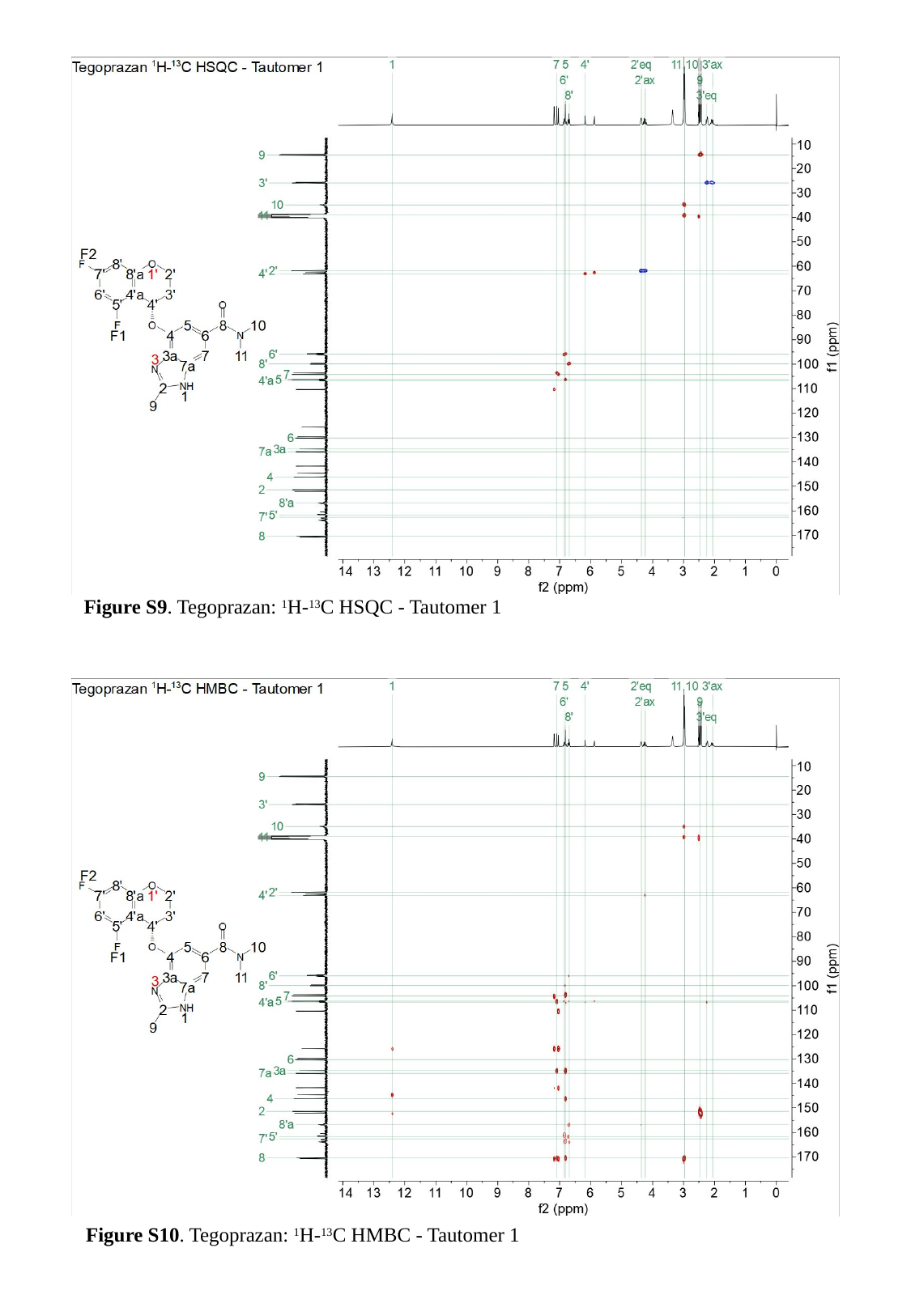

Figure S9. Tegoprazan: 1H-13C HSQC - Tautomer 1
Figure S10. Tegoprazan: 1H-13C HMBC - Tautomer 1

## Slide 9
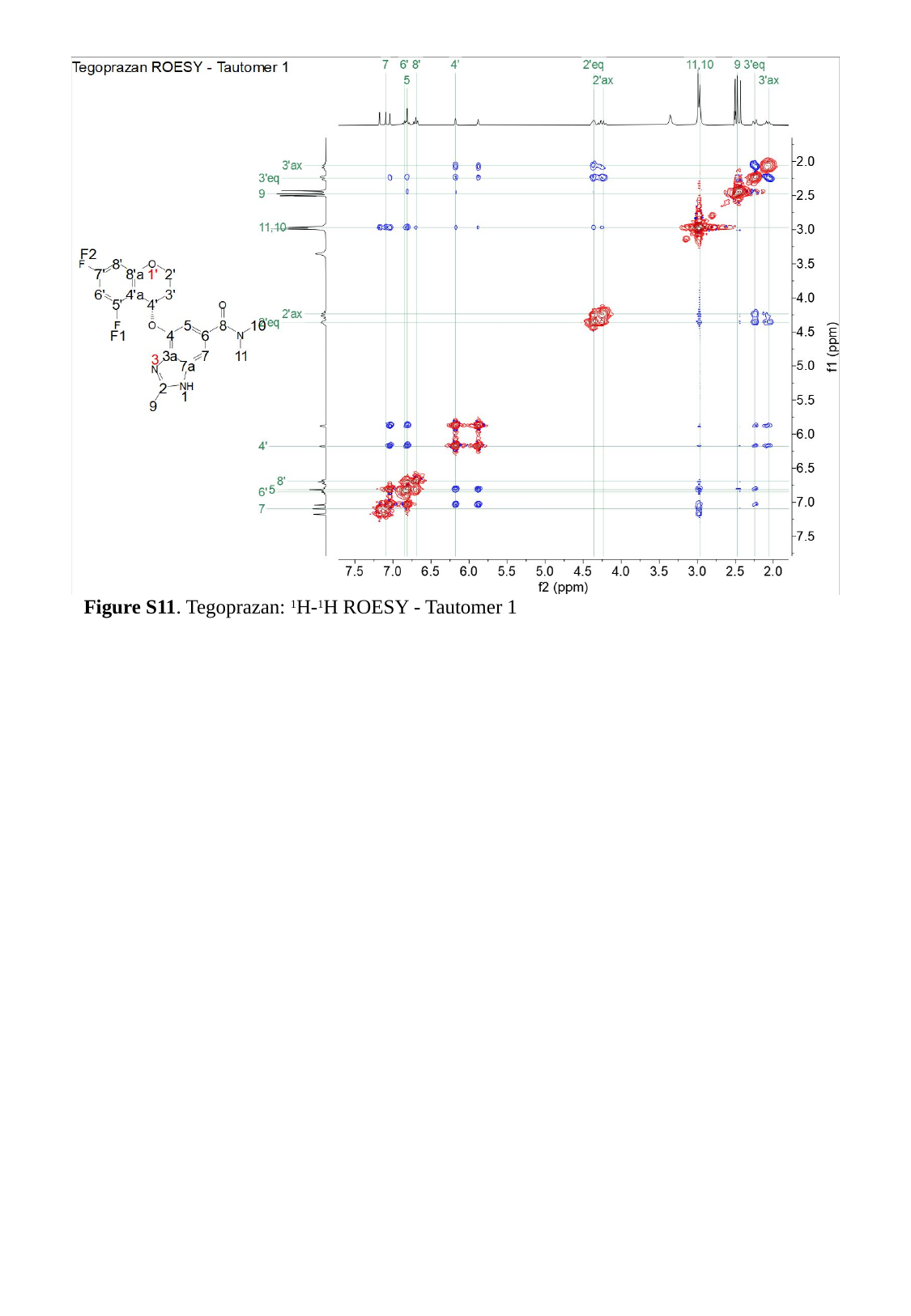

Figure S11. Tegoprazan: 1H-1H ROESY - Tautomer 1

## Slide 10
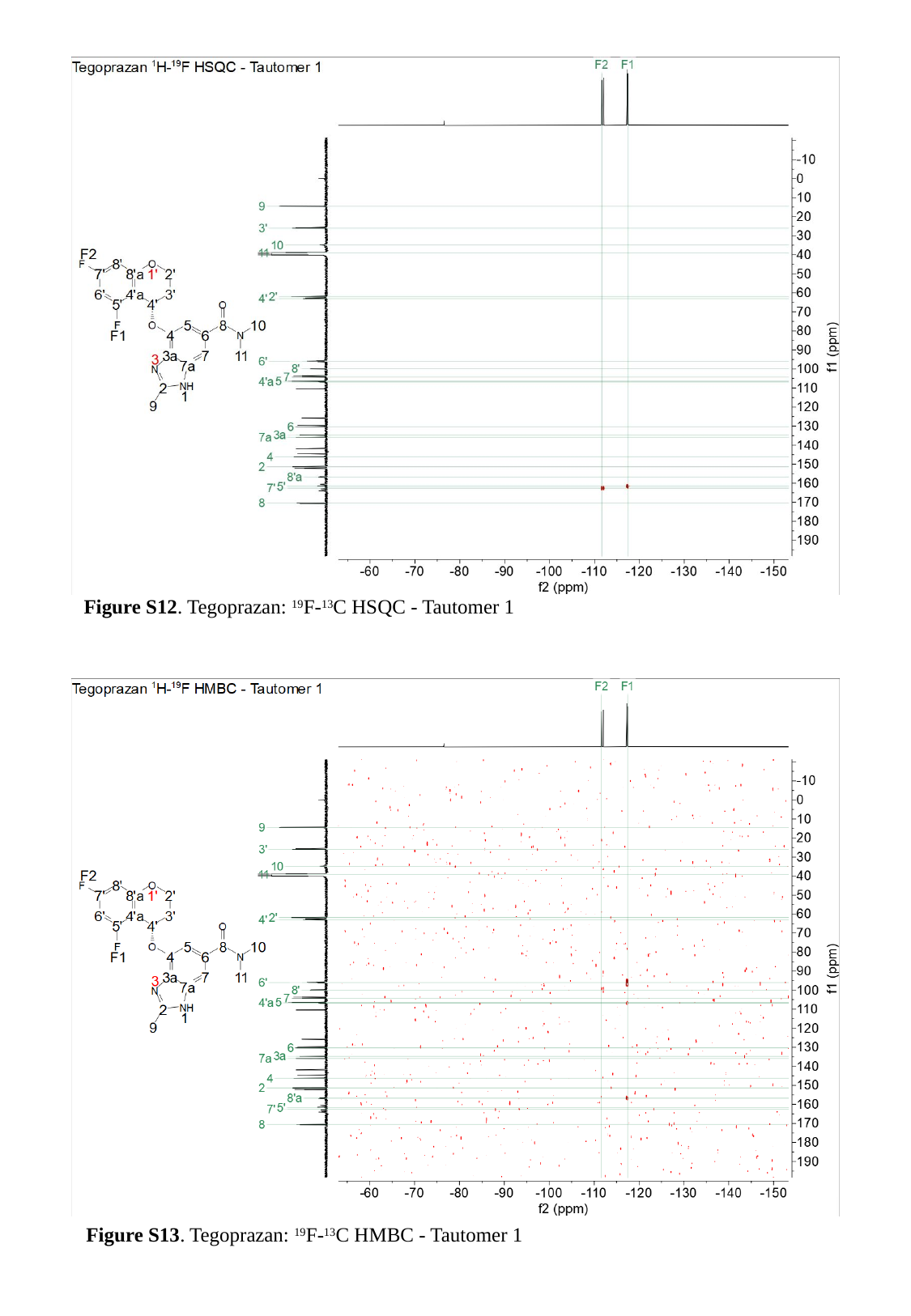

Figure S12. Tegoprazan: 19F-13C HSQC - Tautomer 1
Figure S13. Tegoprazan: 19F-13C HMBC - Tautomer 1

## Slide 11
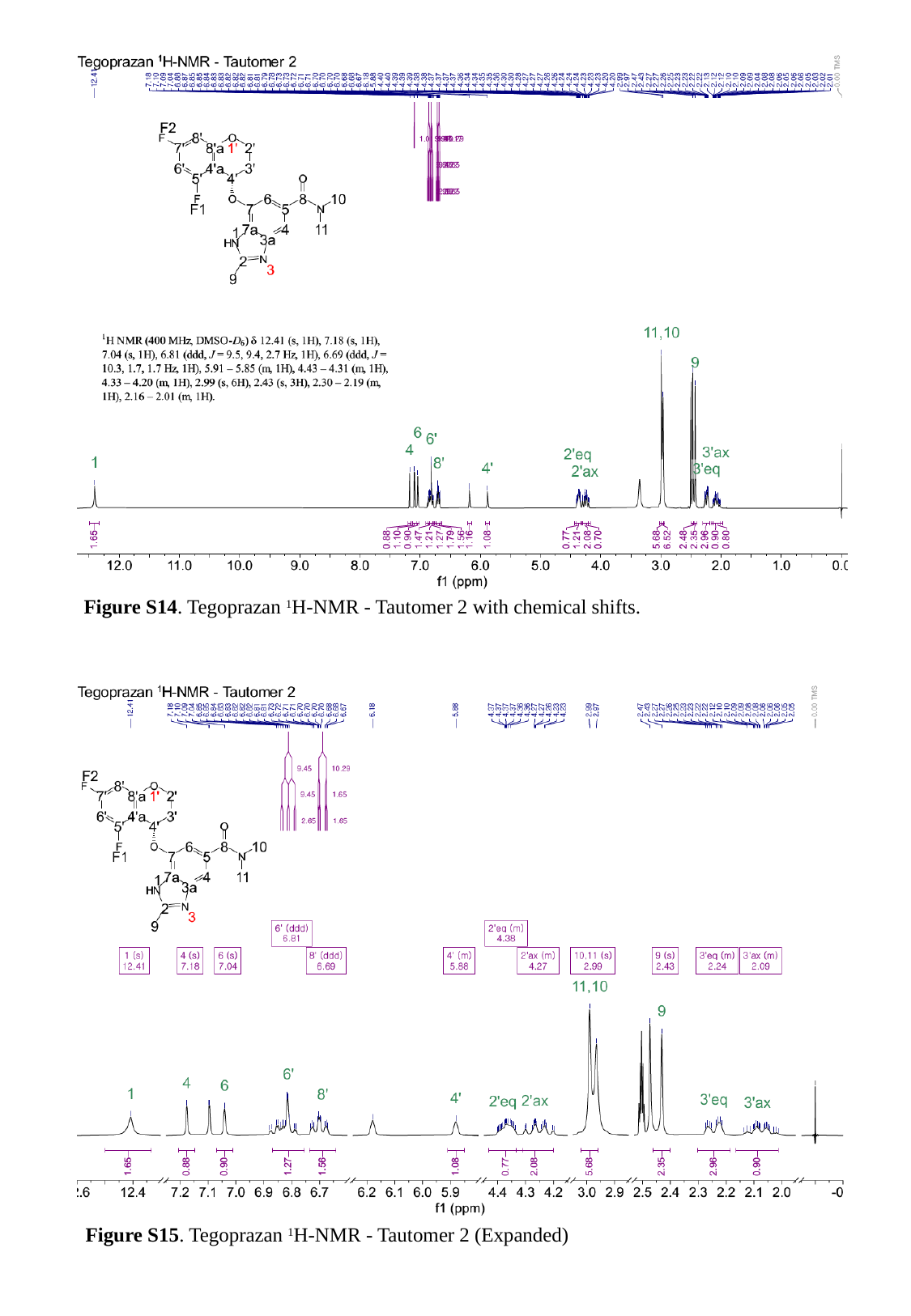

Figure S14. Tegoprazan 1H-NMR - Tautomer 2 with chemical shifts.
Figure S15. Tegoprazan 1H-NMR - Tautomer 2 (Expanded)

## Slide 12
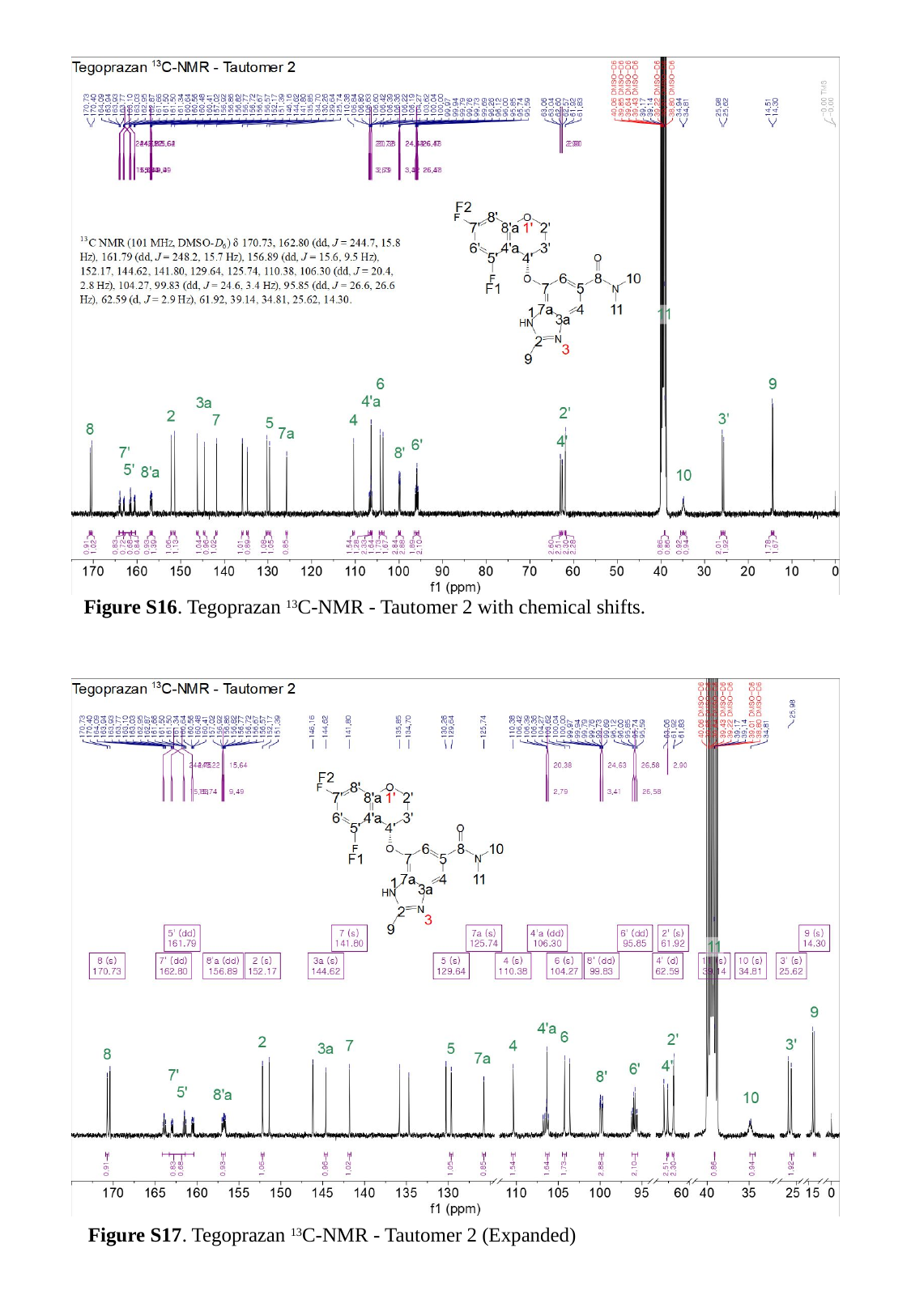

Figure S16. Tegoprazan 13C-NMR - Tautomer 2 with chemical shifts.
Figure S17. Tegoprazan 13C-NMR - Tautomer 2 (Expanded)

## Slide 13
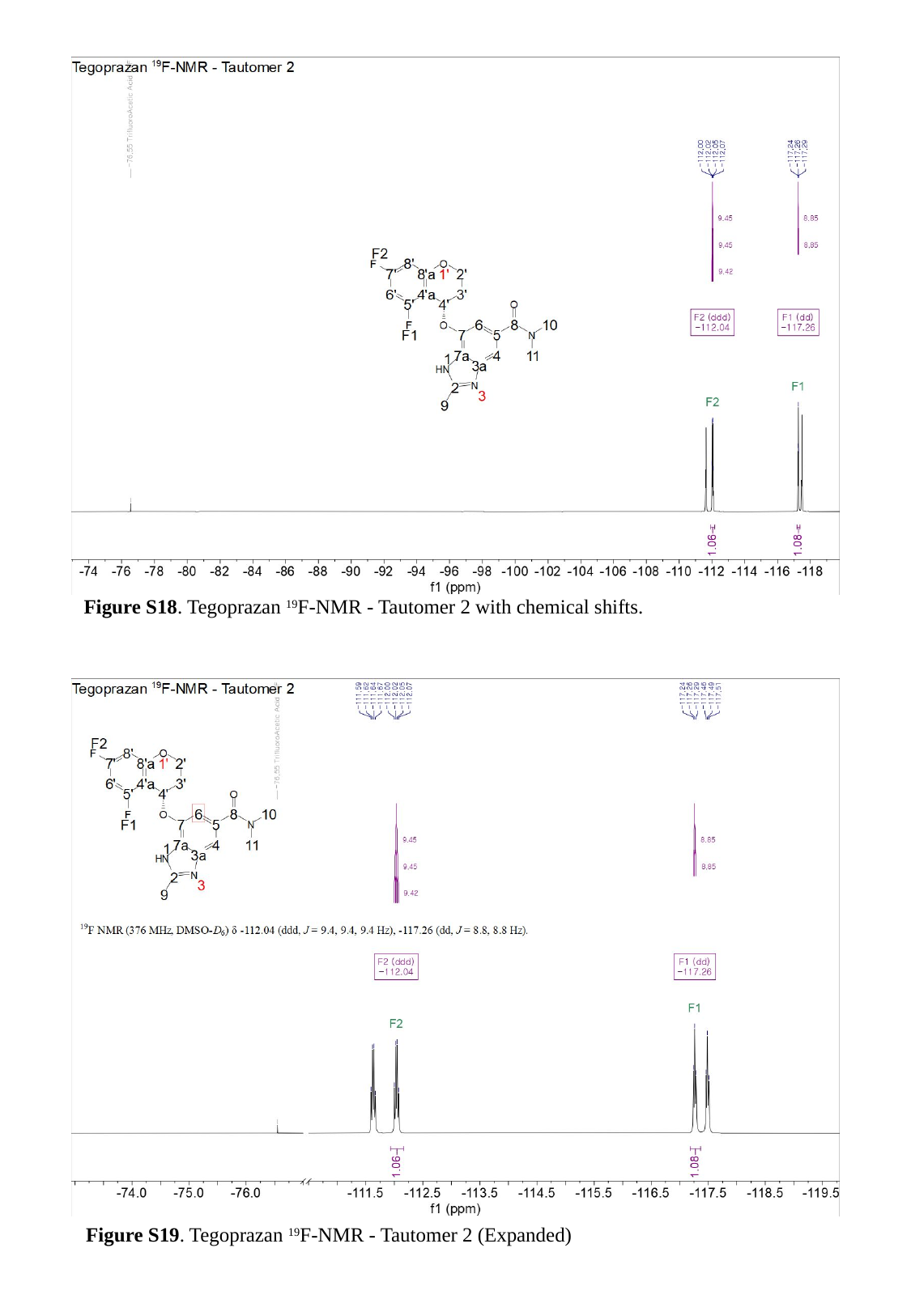

Figure S18. Tegoprazan 19F-NMR - Tautomer 2 with chemical shifts.
Figure S19. Tegoprazan 19F-NMR - Tautomer 2 (Expanded)

## Slide 14
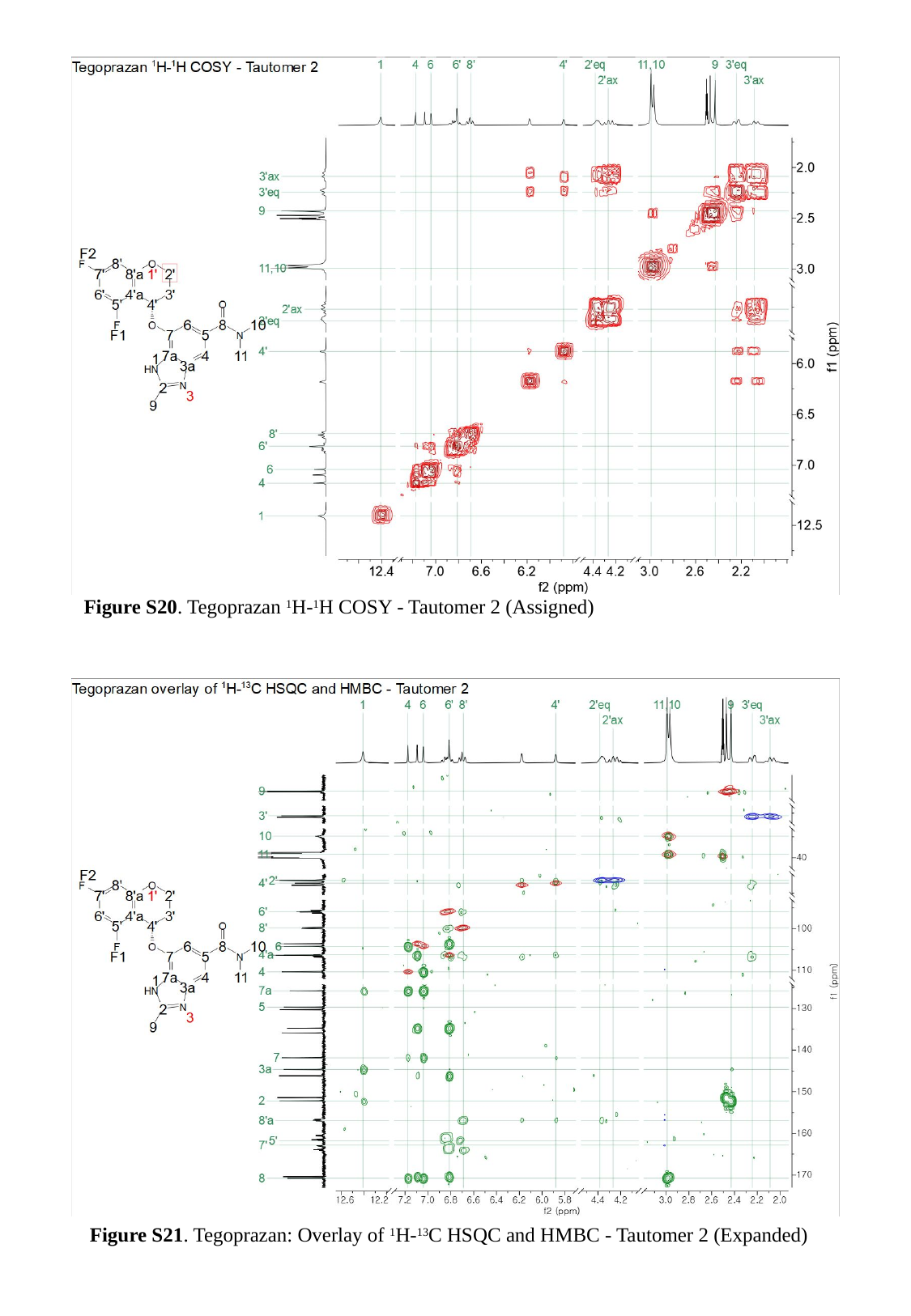

Figure S20. Tegoprazan 1H-1H COSY - Tautomer 2 (Assigned)
Figure S21. Tegoprazan: Overlay of 1H-13C HSQC and HMBC - Tautomer 2 (Expanded)

## Slide 15
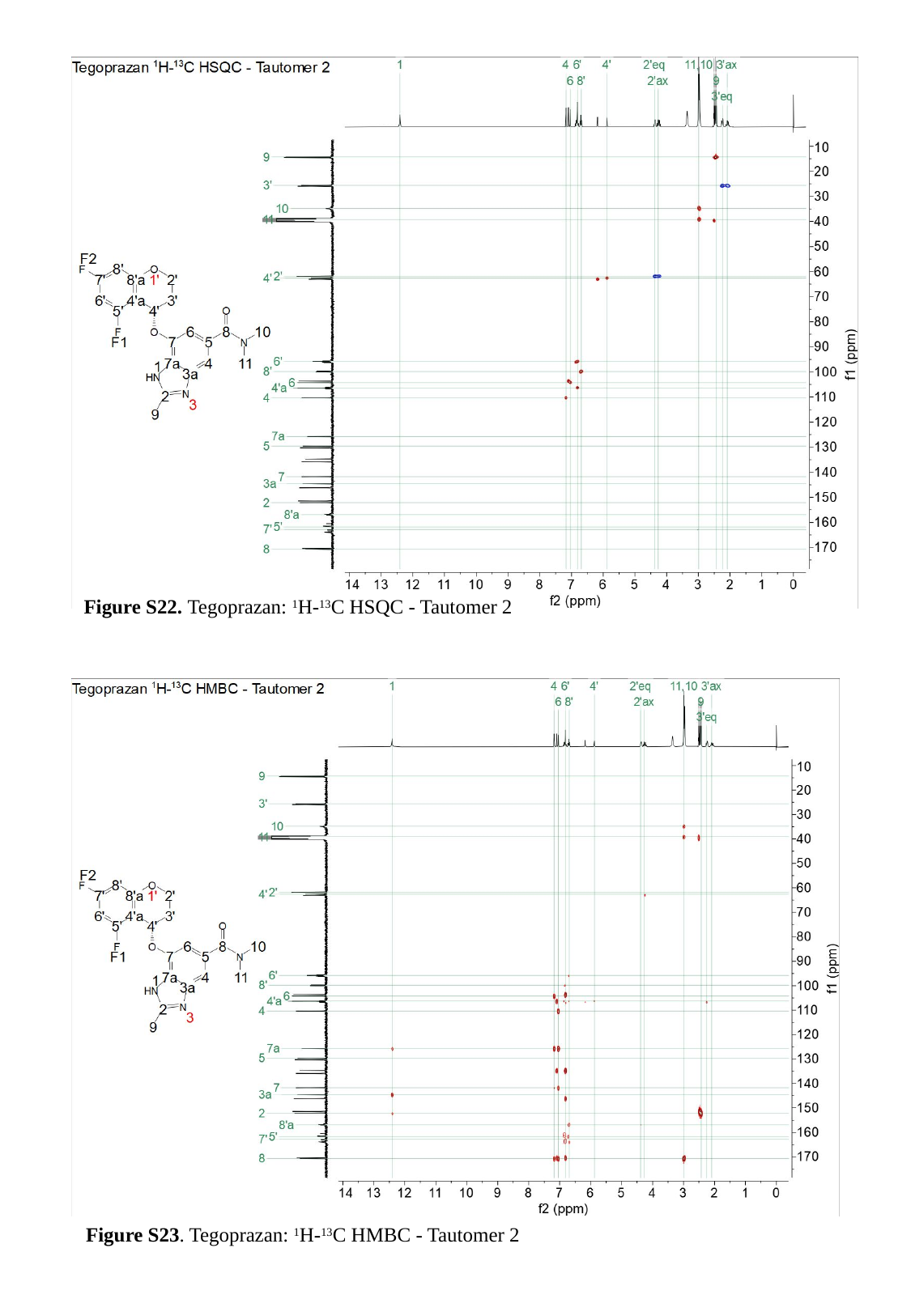

Figure S22. Tegoprazan: 1H-13C HSQC - Tautomer 2
Figure S23. Tegoprazan: 1H-13C HMBC - Tautomer 2

## Slide 16
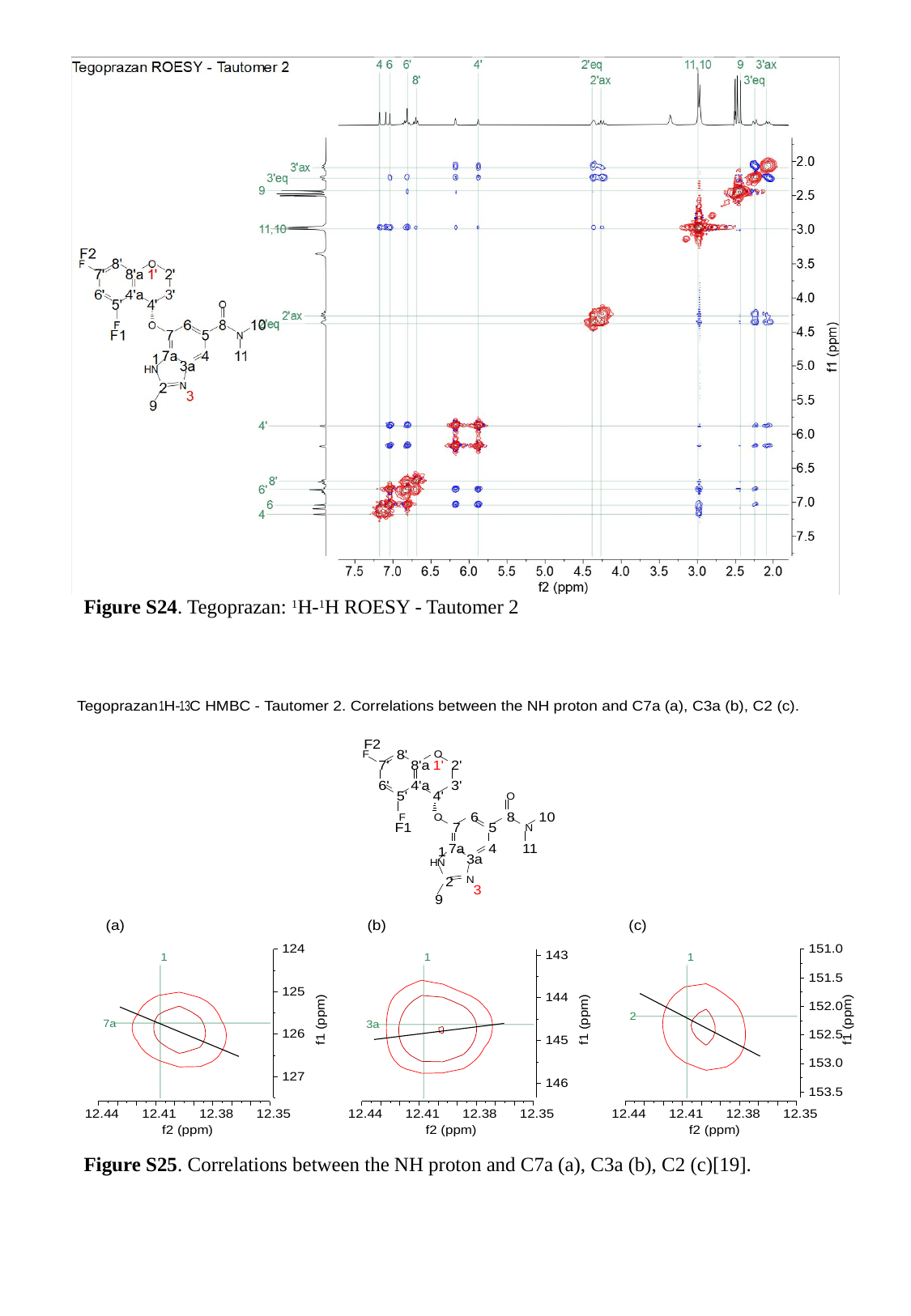

Figure S24. Tegoprazan: 1H-1H ROESY - Tautomer 2
Figure S25. Correlations between the NH proton and C7a (a), C3a (b), C2 (c)[19].

## Slide 17
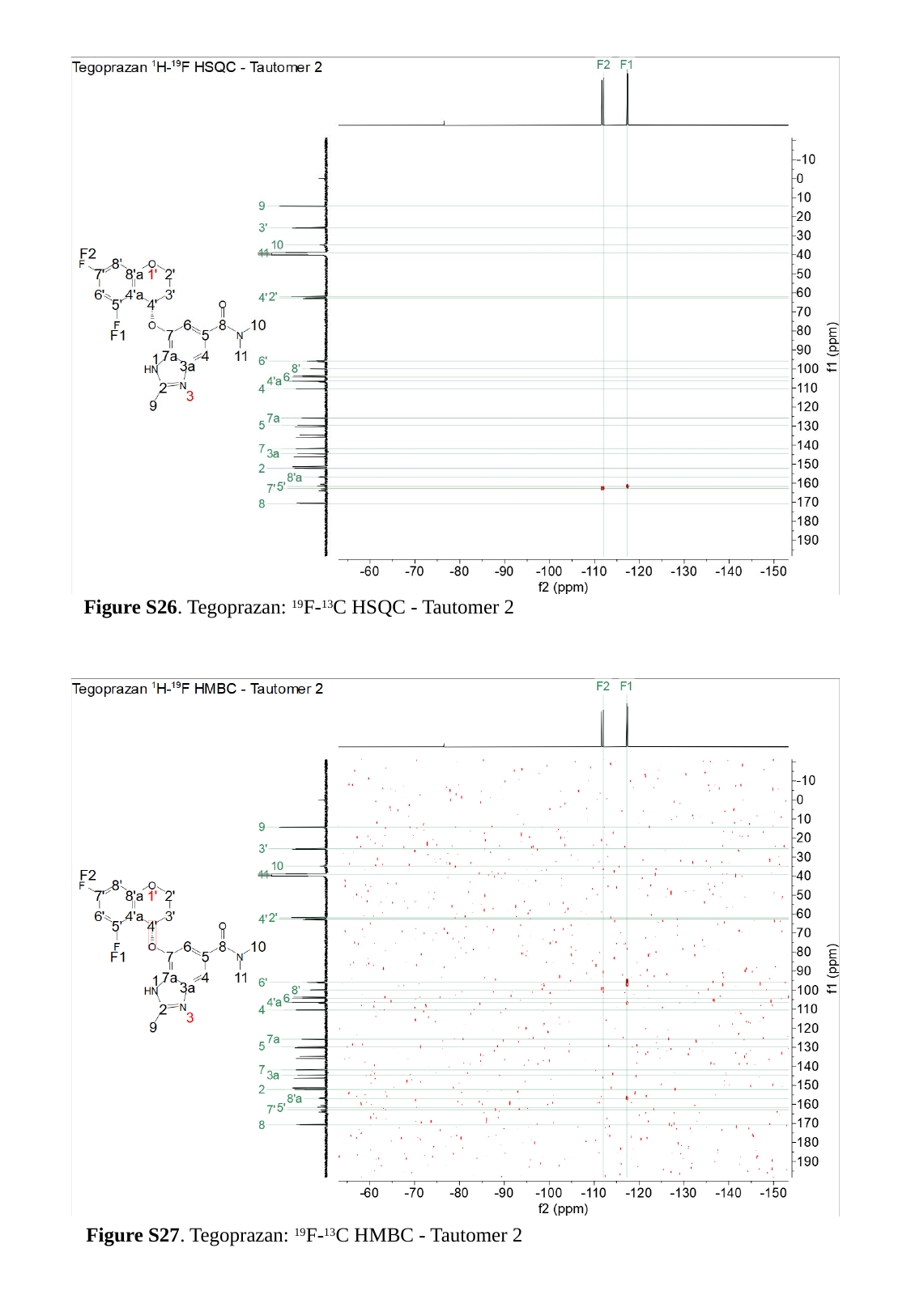

Figure S26. Tegoprazan: 19F-13C HSQC - Tautomer 2
Figure S27. Tegoprazan: 19F-13C HMBC - Tautomer 2

## Slide 18
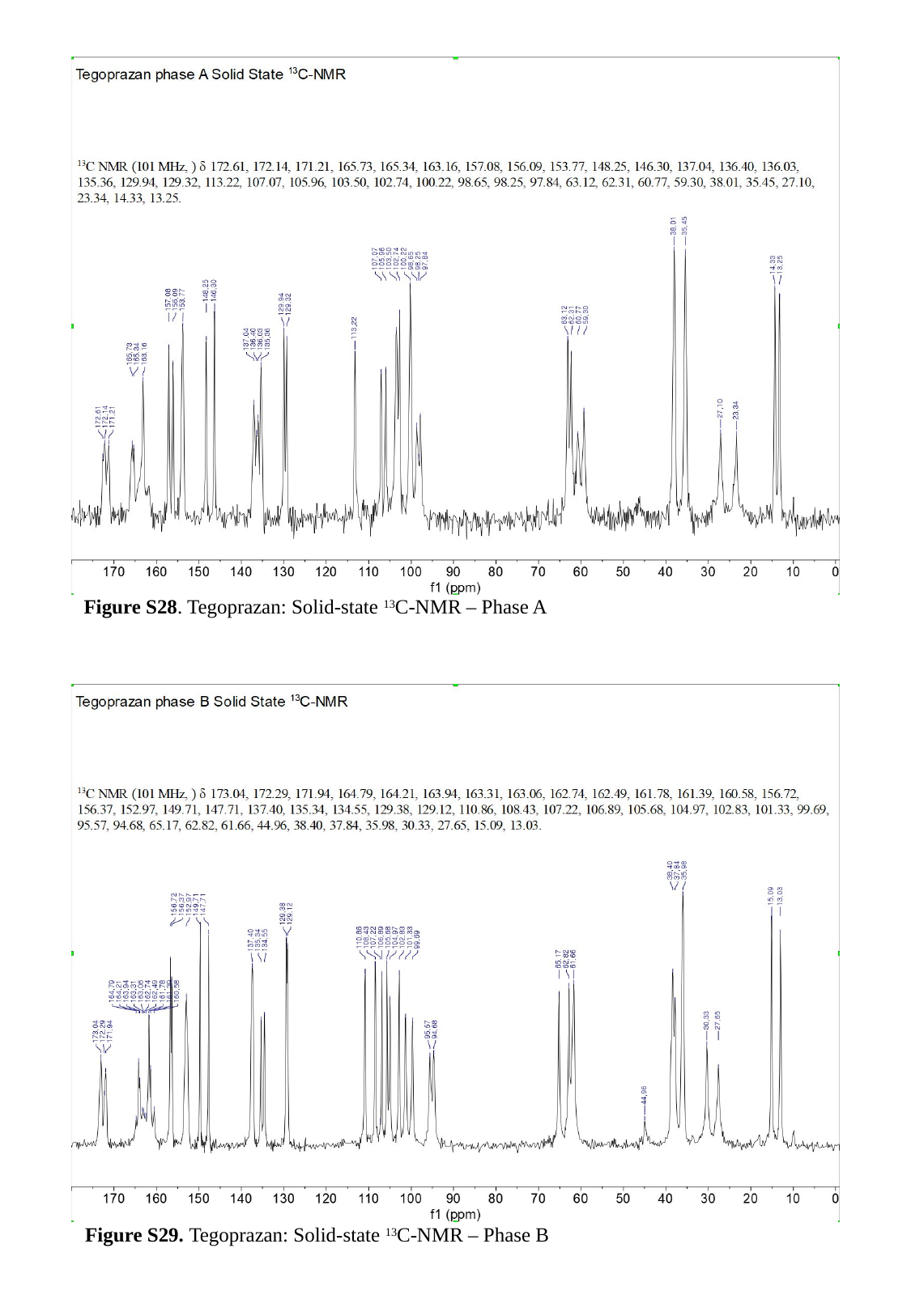

Figure S28. Tegoprazan: Solid-state 13C-NMR – Phase A
Figure S29. Tegoprazan: Solid-state 13C-NMR – Phase B

## Slide 19
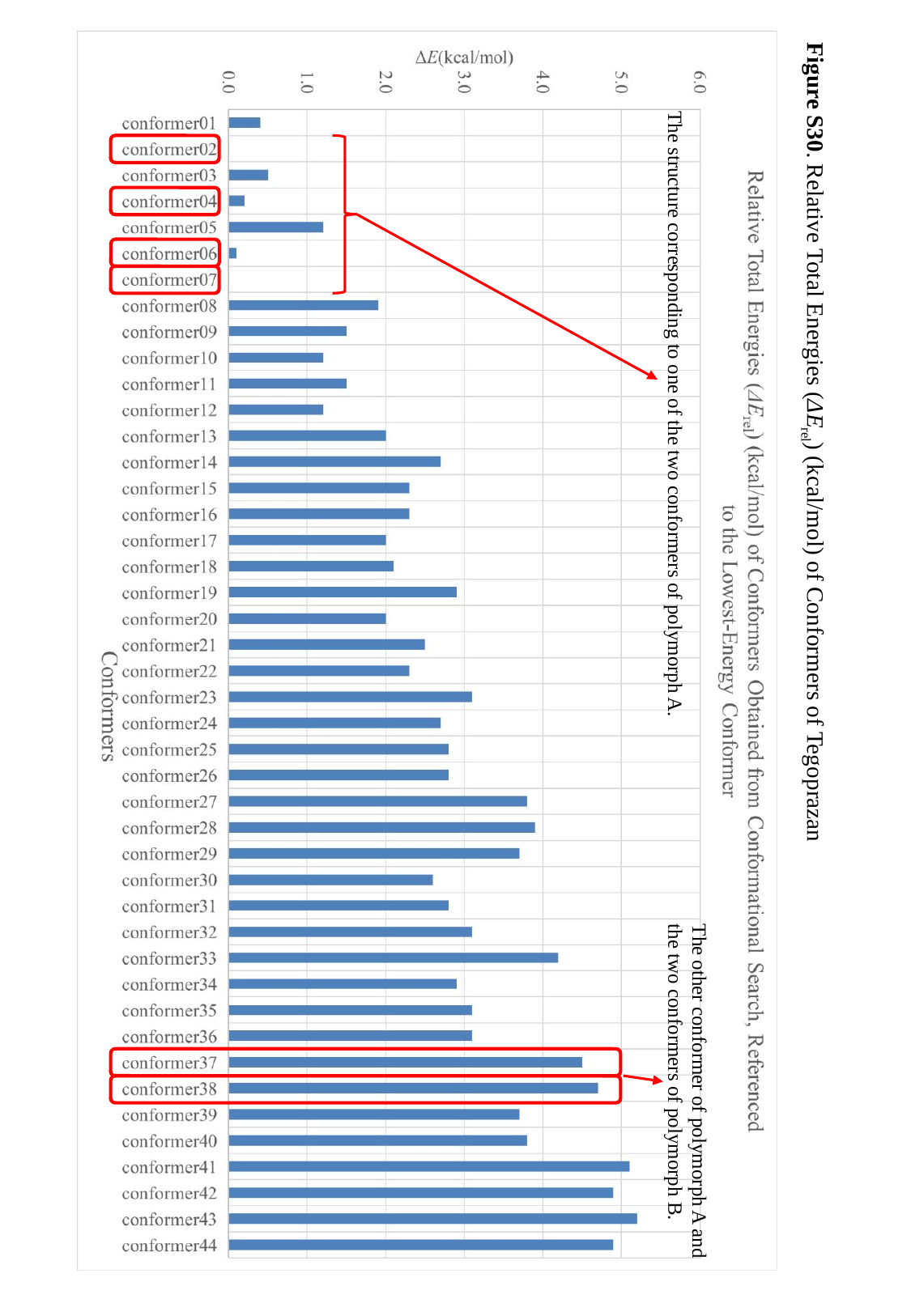

The structure corresponding to one of the two conformers of polymorph A.
Figure S30. Relative Total Energies (ΔErel) (kcal/mol) of Conformers of Tegoprazan
The other conformer of polymorph A and the two conformers of polymorph B.

## Slide 20
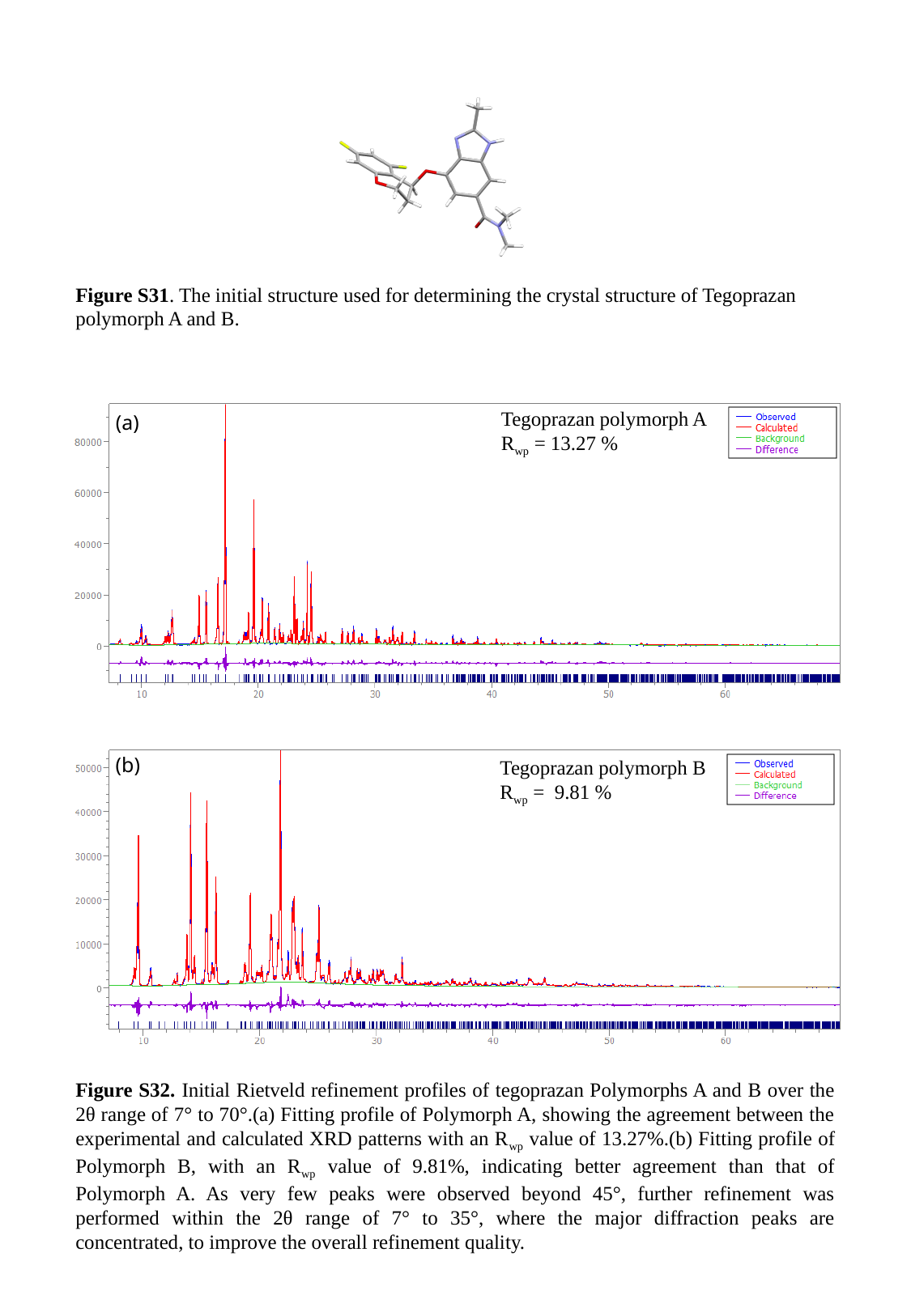

Figure S31. The initial structure used for determining the crystal structure of Tegoprazan polymorph A and B.
Tegoprazan polymorph A
Rwp = 13.27 %
(a)
(b)
Tegoprazan polymorph B
Rwp = 9.81 %
Figure S32. Initial Rietveld refinement profiles of tegoprazan Polymorphs A and B over the 2θ range of 7° to 70°.(a) Fitting profile of Polymorph A, showing the agreement between the experimental and calculated XRD patterns with an Rwp value of 13.27%.(b) Fitting profile of Polymorph B, with an Rwp value of 9.81%, indicating better agreement than that of Polymorph A. As very few peaks were observed beyond 45°, further refinement was performed within the 2θ range of 7° to 35°, where the major diffraction peaks are concentrated, to improve the overall refinement quality.

## Slide 21
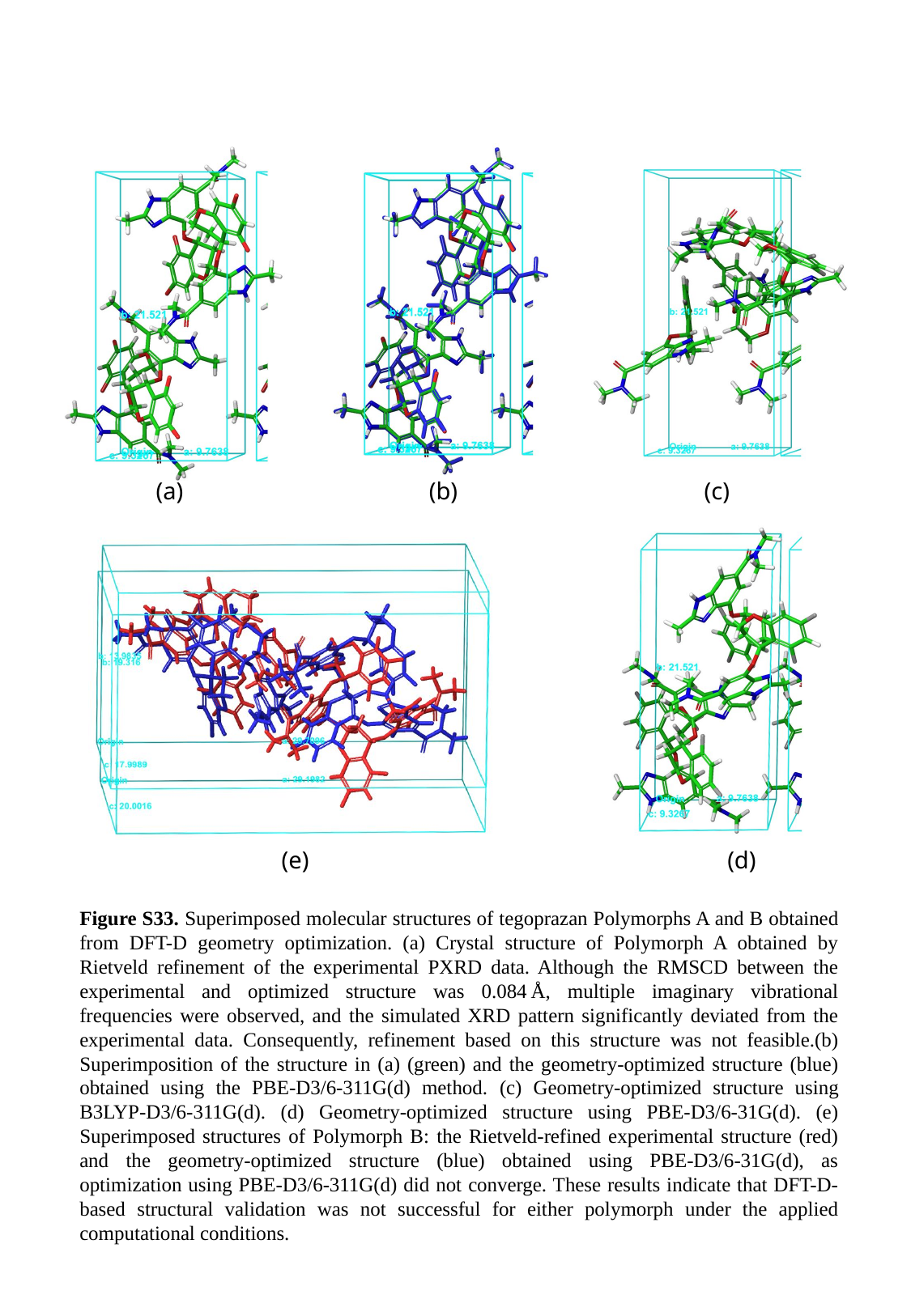

(a)
(b)
(c)
(e)
(d)
Figure S33. Superimposed molecular structures of tegoprazan Polymorphs A and B obtained from DFT-D geometry optimization. (a) Crystal structure of Polymorph A obtained by Rietveld refinement of the experimental PXRD data. Although the RMSCD between the experimental and optimized structure was 0.084 Å, multiple imaginary vibrational frequencies were observed, and the simulated XRD pattern significantly deviated from the experimental data. Consequently, refinement based on this structure was not feasible.(b) Superimposition of the structure in (a) (green) and the geometry-optimized structure (blue) obtained using the PBE-D3/6-311G(d) method. (c) Geometry-optimized structure using B3LYP-D3/6-311G(d). (d) Geometry-optimized structure using PBE-D3/6-31G(d). (e) Superimposed structures of Polymorph B: the Rietveld-refined experimental structure (red) and the geometry-optimized structure (blue) obtained using PBE-D3/6-31G(d), as optimization using PBE-D3/6-311G(d) did not converge. These results indicate that DFT-D-based structural validation was not successful for either polymorph under the applied computational conditions.

## Slide 22
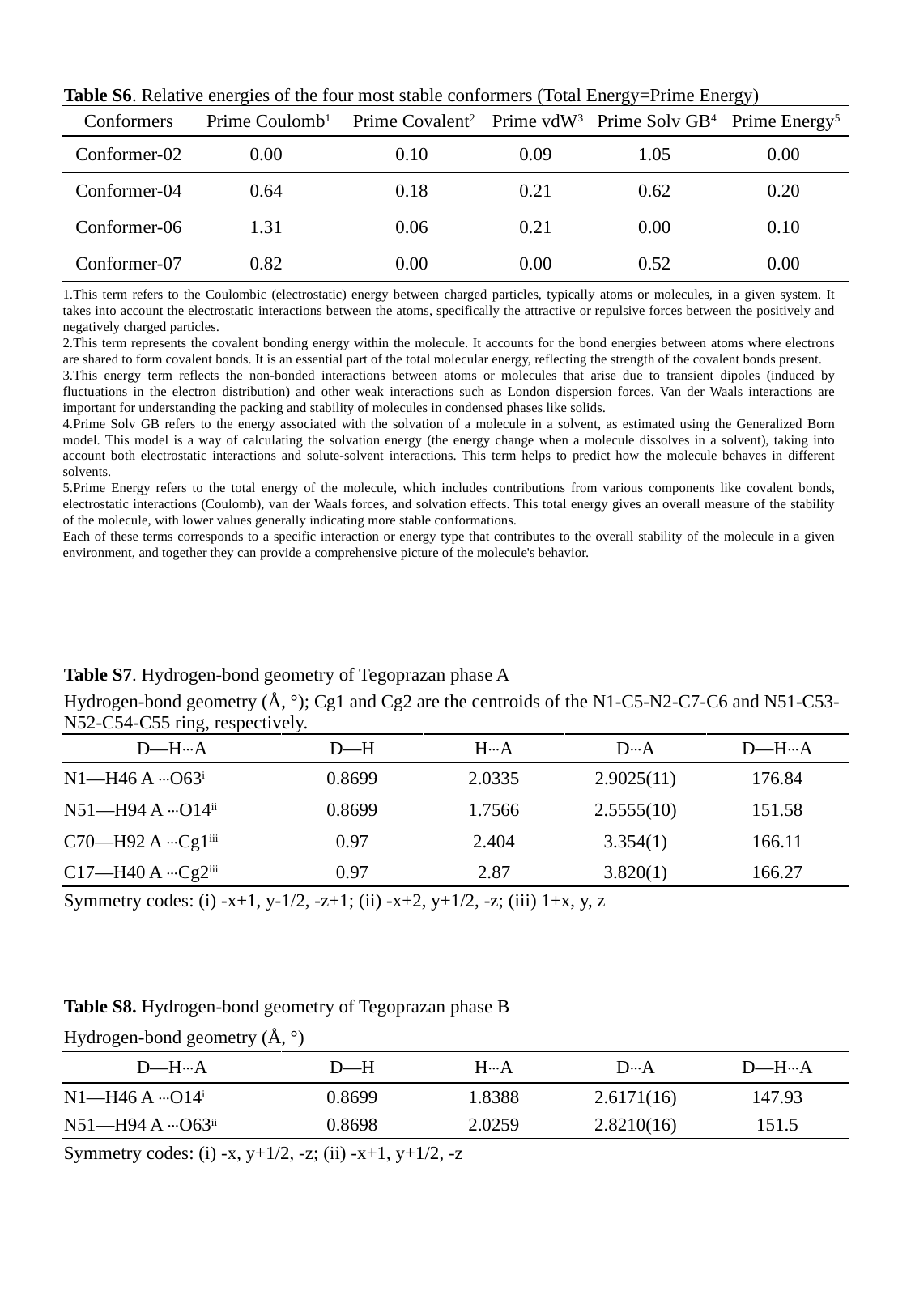

| Table S6. Relative energies of the four most stable conformers (Total Energy=Prime Energy) | | | | | |
| --- | --- | --- | --- | --- | --- |
| Conformers | Prime Coulomb1 | Prime Covalent2 | Prime vdW3 | Prime Solv GB4 | Prime Energy5 |
| Conformer-02 | 0.00 | 0.10 | 0.09 | 1.05 | 0.00 |
| Conformer-04 | 0.64 | 0.18 | 0.21 | 0.62 | 0.20 |
| Conformer-06 | 1.31 | 0.06 | 0.21 | 0.00 | 0.10 |
| Conformer-07 | 0.82 | 0.00 | 0.00 | 0.52 | 0.00 |
This term refers to the Coulombic (electrostatic) energy between charged particles, typically atoms or molecules, in a given system. It takes into account the electrostatic interactions between the atoms, specifically the attractive or repulsive forces between the positively and negatively charged particles.
This term represents the covalent bonding energy within the molecule. It accounts for the bond energies between atoms where electrons are shared to form covalent bonds. It is an essential part of the total molecular energy, reflecting the strength of the covalent bonds present.
This energy term reflects the non-bonded interactions between atoms or molecules that arise due to transient dipoles (induced by fluctuations in the electron distribution) and other weak interactions such as London dispersion forces. Van der Waals interactions are important for understanding the packing and stability of molecules in condensed phases like solids.
Prime Solv GB refers to the energy associated with the solvation of a molecule in a solvent, as estimated using the Generalized Born model. This model is a way of calculating the solvation energy (the energy change when a molecule dissolves in a solvent), taking into account both electrostatic interactions and solute-solvent interactions. This term helps to predict how the molecule behaves in different solvents.
Prime Energy refers to the total energy of the molecule, which includes contributions from various components like covalent bonds, electrostatic interactions (Coulomb), van der Waals forces, and solvation effects. This total energy gives an overall measure of the stability of the molecule, with lower values generally indicating more stable conformations.
Each of these terms corresponds to a specific interaction or energy type that contributes to the overall stability of the molecule in a given environment, and together they can provide a comprehensive picture of the molecule's behavior.
| Table S7. Hydrogen-bond geometry of Tegoprazan phase A | | | | |
| --- | --- | --- | --- | --- |
| Hydrogen-bond geometry (Å, °); Cg1 and Cg2 are the centroids of the N1-C5-N2-C7-C6 and N51-C53-N52-C54-C55 ring, respectively. | | | | |
| D—H⋯A | D—H | H⋯A | D⋯A | D—H⋯A |
| N1—H46 A ⋯O63i | 0.8699 | 2.0335 | 2.9025(11) | 176.84 |
| N51—H94 A ⋯O14ii | 0.8699 | 1.7566 | 2.5555(10) | 151.58 |
| C70—H92 A ⋯Cg1iii | 0.97 | 2.404 | 3.354(1) | 166.11 |
| C17—H40 A ⋯Cg2iii | 0.97 | 2.87 | 3.820(1) | 166.27 |
| Symmetry codes: (i) -x+1, y-1/2, -z+1; (ii) -x+2, y+1/2, -z; (iii) 1+x, y, z | | | | |
| Table S8. Hydrogen-bond geometry of Tegoprazan phase B | | | | |
| --- | --- | --- | --- | --- |
| Hydrogen-bond geometry (Å, °) | | | | |
| D—H⋯A | D—H | H⋯A | D⋯A | D—H⋯A |
| N1—H46 A ⋯O14i | 0.8699 | 1.8388 | 2.6171(16) | 147.93 |
| N51—H94 A ⋯O63ii | 0.8698 | 2.0259 | 2.8210(16) | 151.5 |
| Symmetry codes: (i) -x, y+1/2, -z; (ii) -x+1, y+1/2, -z | | | | |

## Slide 23
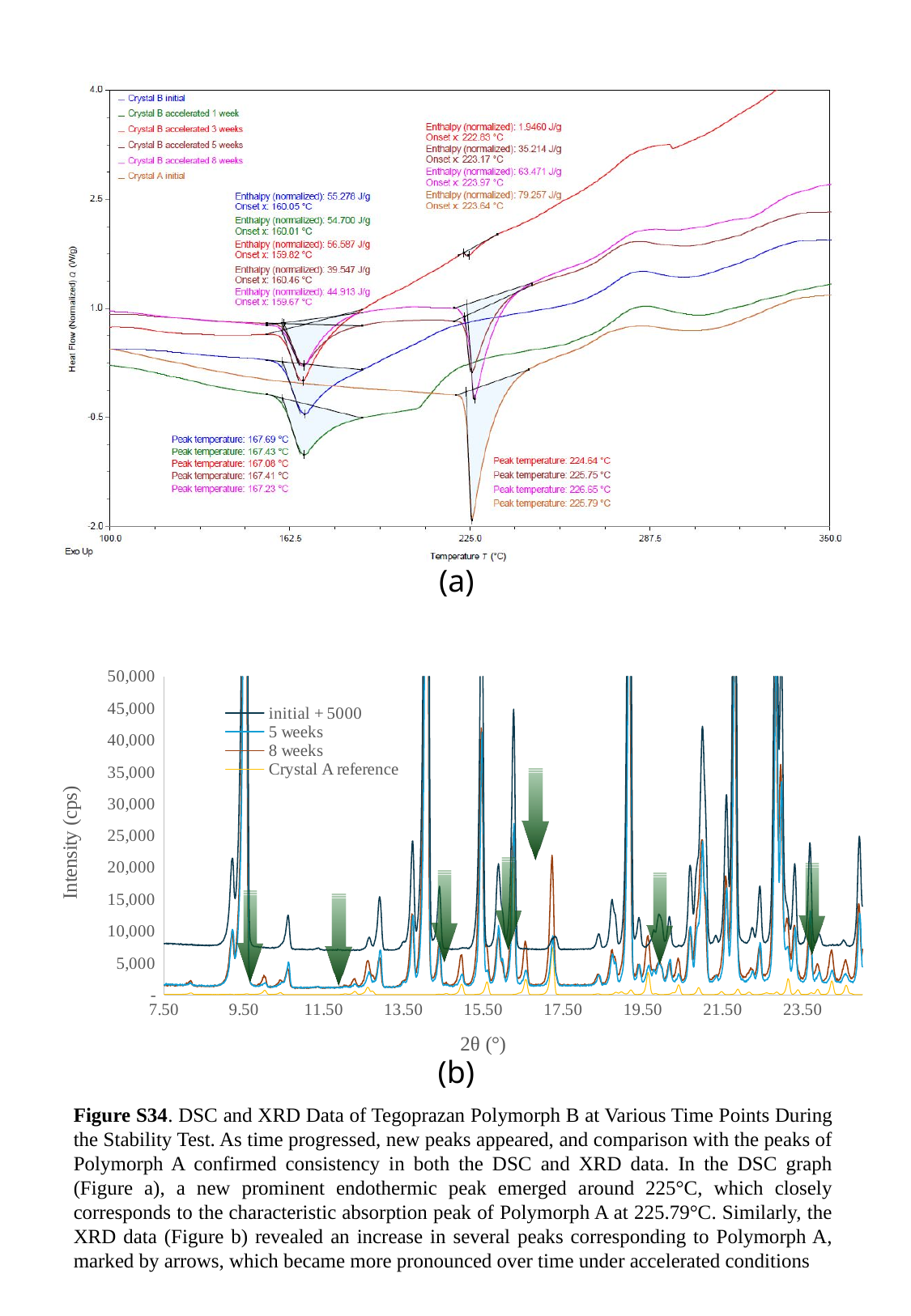

(a)
### Chart
| Category | initial + 5000 | 5 weeks | 8 weeks | Crystal A reference |
|---|---|---|---|---|
(b)
Figure S34. DSC and XRD Data of Tegoprazan Polymorph B at Various Time Points During the Stability Test. As time progressed, new peaks appeared, and comparison with the peaks of Polymorph A confirmed consistency in both the DSC and XRD data. In the DSC graph (Figure a), a new prominent endothermic peak emerged around 225°C, which closely corresponds to the characteristic absorption peak of Polymorph A at 225.79°C. Similarly, the XRD data (Figure b) revealed an increase in several peaks corresponding to Polymorph A, marked by arrows, which became more pronounced over time under accelerated conditions

## Slide 24
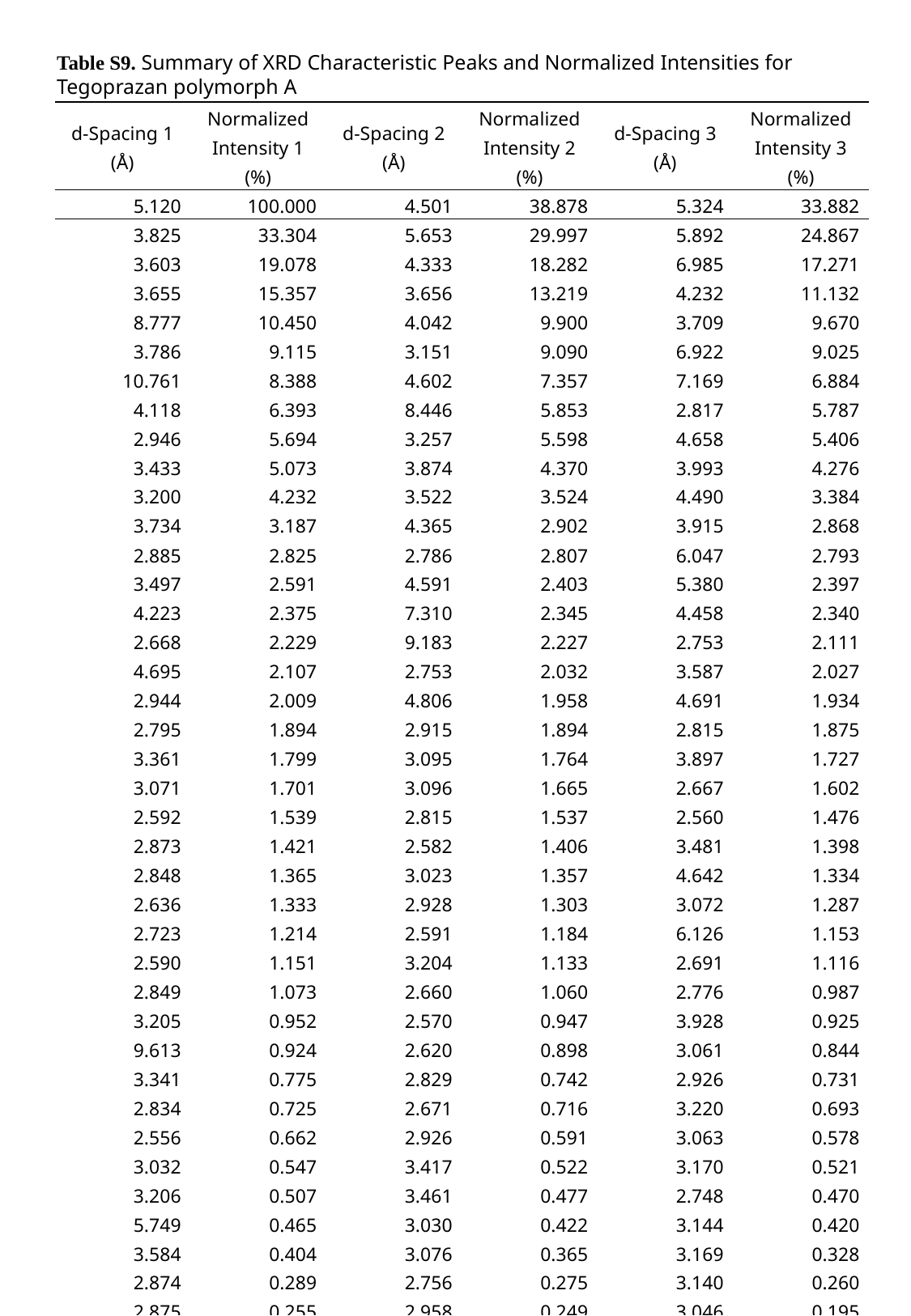

Table S9. Summary of XRD Characteristic Peaks and Normalized Intensities for Tegoprazan polymorph A
| d-Spacing 1 (Å) | Normalized Intensity 1 (%) | d-Spacing 2 (Å) | Normalized Intensity 2 (%) | d-Spacing 3 (Å) | Normalized Intensity 3 (%) |
| --- | --- | --- | --- | --- | --- |
| 5.120 | 100.000 | 4.501 | 38.878 | 5.324 | 33.882 |
| 3.825 | 33.304 | 5.653 | 29.997 | 5.892 | 24.867 |
| 3.603 | 19.078 | 4.333 | 18.282 | 6.985 | 17.271 |
| 3.655 | 15.357 | 3.656 | 13.219 | 4.232 | 11.132 |
| 8.777 | 10.450 | 4.042 | 9.900 | 3.709 | 9.670 |
| 3.786 | 9.115 | 3.151 | 9.090 | 6.922 | 9.025 |
| 10.761 | 8.388 | 4.602 | 7.357 | 7.169 | 6.884 |
| 4.118 | 6.393 | 8.446 | 5.853 | 2.817 | 5.787 |
| 2.946 | 5.694 | 3.257 | 5.598 | 4.658 | 5.406 |
| 3.433 | 5.073 | 3.874 | 4.370 | 3.993 | 4.276 |
| 3.200 | 4.232 | 3.522 | 3.524 | 4.490 | 3.384 |
| 3.734 | 3.187 | 4.365 | 2.902 | 3.915 | 2.868 |
| 2.885 | 2.825 | 2.786 | 2.807 | 6.047 | 2.793 |
| 3.497 | 2.591 | 4.591 | 2.403 | 5.380 | 2.397 |
| 4.223 | 2.375 | 7.310 | 2.345 | 4.458 | 2.340 |
| 2.668 | 2.229 | 9.183 | 2.227 | 2.753 | 2.111 |
| 4.695 | 2.107 | 2.753 | 2.032 | 3.587 | 2.027 |
| 2.944 | 2.009 | 4.806 | 1.958 | 4.691 | 1.934 |
| 2.795 | 1.894 | 2.915 | 1.894 | 2.815 | 1.875 |
| 3.361 | 1.799 | 3.095 | 1.764 | 3.897 | 1.727 |
| 3.071 | 1.701 | 3.096 | 1.665 | 2.667 | 1.602 |
| 2.592 | 1.539 | 2.815 | 1.537 | 2.560 | 1.476 |
| 2.873 | 1.421 | 2.582 | 1.406 | 3.481 | 1.398 |
| 2.848 | 1.365 | 3.023 | 1.357 | 4.642 | 1.334 |
| 2.636 | 1.333 | 2.928 | 1.303 | 3.072 | 1.287 |
| 2.723 | 1.214 | 2.591 | 1.184 | 6.126 | 1.153 |
| 2.590 | 1.151 | 3.204 | 1.133 | 2.691 | 1.116 |
| 2.849 | 1.073 | 2.660 | 1.060 | 2.776 | 0.987 |
| 3.205 | 0.952 | 2.570 | 0.947 | 3.928 | 0.925 |
| 9.613 | 0.924 | 2.620 | 0.898 | 3.061 | 0.844 |
| 3.341 | 0.775 | 2.829 | 0.742 | 2.926 | 0.731 |
| 2.834 | 0.725 | 2.671 | 0.716 | 3.220 | 0.693 |
| 2.556 | 0.662 | 2.926 | 0.591 | 3.063 | 0.578 |
| 3.032 | 0.547 | 3.417 | 0.522 | 3.170 | 0.521 |
| 3.206 | 0.507 | 3.461 | 0.477 | 2.748 | 0.470 |
| 5.749 | 0.465 | 3.030 | 0.422 | 3.144 | 0.420 |
| 3.584 | 0.404 | 3.076 | 0.365 | 3.169 | 0.328 |
| 2.874 | 0.289 | 2.756 | 0.275 | 3.140 | 0.260 |
| 2.875 | 0.255 | 2.958 | 0.249 | 3.046 | 0.195 |
| 4.389 | 0.158 | 2.665 | 0.135 | 3.492 | 0.118 |
| 2.571 | 0.098 | 2.923 | 0.094 | 3.867 | 0.092 |
| 2.690 | 0.089 | 3.887 | 0.082 | 2.777 | 0.073 |
| 2.779 | 0.069 | 2.827 | 0.067 | 2.827 | 0.039 |
| 2.791 | 0.008 | 2.555 | 0.008 | 2.662 | 0.007 |
| 3.982 | 0.002 | | | | |

## Slide 25
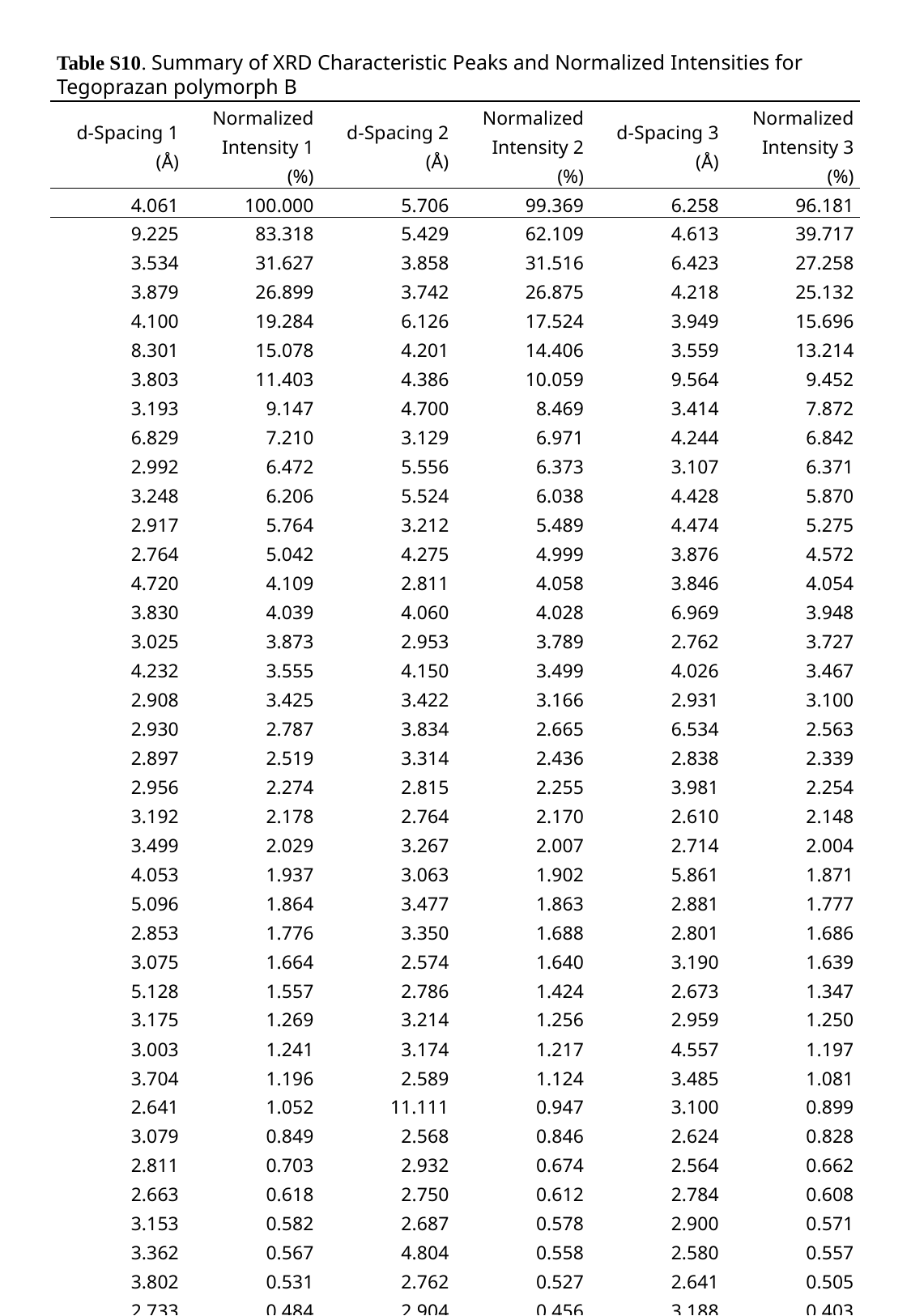

Table S10. Summary of XRD Characteristic Peaks and Normalized Intensities for Tegoprazan polymorph B
| d-Spacing 1 (Å) | Normalized Intensity 1 (%) | d-Spacing 2 (Å) | Normalized Intensity 2 (%) | d-Spacing 3 (Å) | Normalized Intensity 3 (%) |
| --- | --- | --- | --- | --- | --- |
| 4.061 | 100.000 | 5.706 | 99.369 | 6.258 | 96.181 |
| 9.225 | 83.318 | 5.429 | 62.109 | 4.613 | 39.717 |
| 3.534 | 31.627 | 3.858 | 31.516 | 6.423 | 27.258 |
| 3.879 | 26.899 | 3.742 | 26.875 | 4.218 | 25.132 |
| 4.100 | 19.284 | 6.126 | 17.524 | 3.949 | 15.696 |
| 8.301 | 15.078 | 4.201 | 14.406 | 3.559 | 13.214 |
| 3.803 | 11.403 | 4.386 | 10.059 | 9.564 | 9.452 |
| 3.193 | 9.147 | 4.700 | 8.469 | 3.414 | 7.872 |
| 6.829 | 7.210 | 3.129 | 6.971 | 4.244 | 6.842 |
| 2.992 | 6.472 | 5.556 | 6.373 | 3.107 | 6.371 |
| 3.248 | 6.206 | 5.524 | 6.038 | 4.428 | 5.870 |
| 2.917 | 5.764 | 3.212 | 5.489 | 4.474 | 5.275 |
| 2.764 | 5.042 | 4.275 | 4.999 | 3.876 | 4.572 |
| 4.720 | 4.109 | 2.811 | 4.058 | 3.846 | 4.054 |
| 3.830 | 4.039 | 4.060 | 4.028 | 6.969 | 3.948 |
| 3.025 | 3.873 | 2.953 | 3.789 | 2.762 | 3.727 |
| 4.232 | 3.555 | 4.150 | 3.499 | 4.026 | 3.467 |
| 2.908 | 3.425 | 3.422 | 3.166 | 2.931 | 3.100 |
| 2.930 | 2.787 | 3.834 | 2.665 | 6.534 | 2.563 |
| 2.897 | 2.519 | 3.314 | 2.436 | 2.838 | 2.339 |
| 2.956 | 2.274 | 2.815 | 2.255 | 3.981 | 2.254 |
| 3.192 | 2.178 | 2.764 | 2.170 | 2.610 | 2.148 |
| 3.499 | 2.029 | 3.267 | 2.007 | 2.714 | 2.004 |
| 4.053 | 1.937 | 3.063 | 1.902 | 5.861 | 1.871 |
| 5.096 | 1.864 | 3.477 | 1.863 | 2.881 | 1.777 |
| 2.853 | 1.776 | 3.350 | 1.688 | 2.801 | 1.686 |
| 3.075 | 1.664 | 2.574 | 1.640 | 3.190 | 1.639 |
| 5.128 | 1.557 | 2.786 | 1.424 | 2.673 | 1.347 |
| 3.175 | 1.269 | 3.214 | 1.256 | 2.959 | 1.250 |
| 3.003 | 1.241 | 3.174 | 1.217 | 4.557 | 1.197 |
| 3.704 | 1.196 | 2.589 | 1.124 | 3.485 | 1.081 |
| 2.641 | 1.052 | 11.111 | 0.947 | 3.100 | 0.899 |
| 3.079 | 0.849 | 2.568 | 0.846 | 2.624 | 0.828 |
| 2.811 | 0.703 | 2.932 | 0.674 | 2.564 | 0.662 |
| 2.663 | 0.618 | 2.750 | 0.612 | 2.784 | 0.608 |
| 3.153 | 0.582 | 2.687 | 0.578 | 2.900 | 0.571 |
| 3.362 | 0.567 | 4.804 | 0.558 | 2.580 | 0.557 |
| 3.802 | 0.531 | 2.762 | 0.527 | 2.641 | 0.505 |
| 2.733 | 0.484 | 2.904 | 0.456 | 3.188 | 0.403 |
| 4.557 | 0.401 | 2.848 | 0.397 | 2.589 | 0.383 |
| 3.640 | 0.375 | 3.613 | 0.372 | 2.767 | 0.356 |
| 3.091 | 0.319 | 2.965 | 0.316 | 4.444 | 0.312 |
| 2.586 | 0.306 | 3.136 | 0.303 | 2.778 | 0.269 |
| 4.782 | 0.237 | 7.757 | 0.234 | 2.625 | 0.217 |
| 2.628 | 0.216 | 3.274 | 0.195 | 7.407 | 0.181 |
| 2.693 | 0.169 | 2.653 | 0.139 | 2.596 | 0.135 |
| 2.992 | 0.068 | 2.816 | 0.025 | 2.639 | 0.023 |
| 8.403 | 0.016 | 3.486 | 0.010 | 3.065 | 0.001 |

## Slide 26
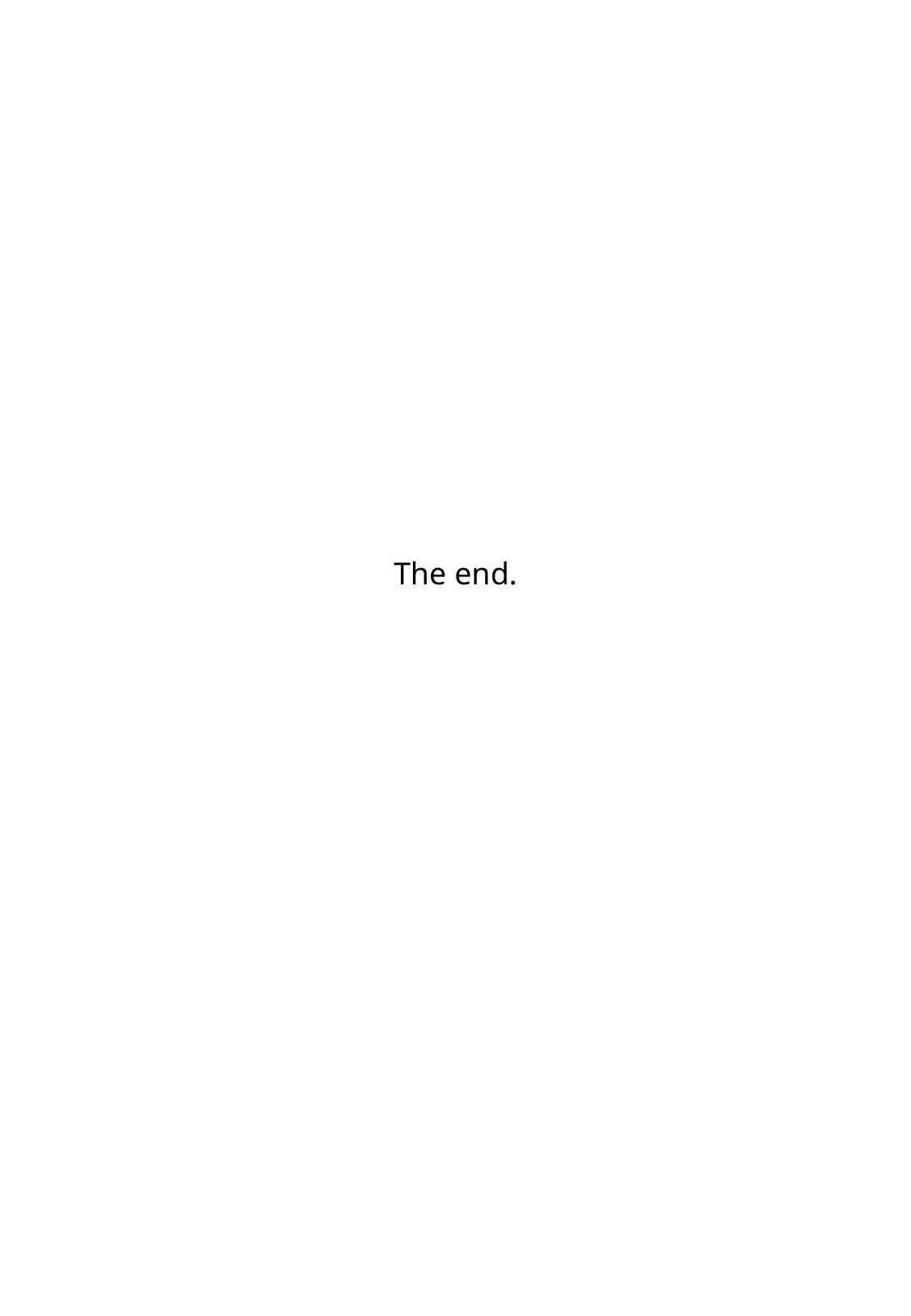

The end.
